# Asparagine Endopeptidase Controls Anti-Influenza Virus Immune Responses through TLR7 Activation

**DOI:** 10.1371/journal.ppat.1002841

**Published:** 2012-08-16

**Authors:** Sophia Maschalidi, Signe Hässler, Fany Blanc, Fernando E. Sepulveda, Mira Tohme, Michel Chignard, Peter van Endert, Mustapha Si-Tahar, Delphyne Descamps, Bénédicte Manoury

**Affiliations:** 1 INSERM, Unité 1013, Paris, France; 2 Université Paris Descartes, Sorbonne Paris Cité, Faculté de Médecine, Paris, France; 3 Unité de Défense Innée et Inflammation, Institut Pasteur, Paris, France; 4 INSERM, Unité 874, Paris, France; 5 INSERM, Unité 768, Paris, France; 6 INSERM, Unité 932, Institut Curie, Paris, France; 7 INSERM, Unité 1100, Tours, France; 8 Faculté de Médecine F. Rabelais, Tours, France; Johns Hopkins University - Bloomberg School of Public Health, United States of America

## Abstract

Intracellular Toll-like receptors (TLRs) expressed by dendritic cells recognize nucleic acids derived from pathogens and play an important role in the immune responses against the influenza virus (IAV), a single-stranded RNA sensed by different receptors including TLR7. However, the importance of TLR7 processing in the development of anti-viral immune responses is not known. Here we report that asparagine endopeptidase (AEP) deficient mice are unable to generate a strong anti-IAV response, as demonstrated by reduced inflammation, cross presentation of cell-associated antigens and priming of CD8^+^ T cells following TLR7-dependent pulmonary infection induced by IAV. Moreover, AEP deficient lung epithelial- or myeloid-cells exhibit impaired TLR7 signaling due to defective processing of this receptor. Indeed, TLR7 requires a proteolytic cleavage by AEP to generate a C-terminal fragment competent for signaling. Thus, AEP activity is critical for TLR7 processing, opening new possibilities for the treatment of influenza and TLR7-dependent inflammatory diseases.

## Author Summary

Influenza A virus, a negative stranded RNA, can cause severe illness in humans and animals and stimulates many receptors including Toll like receptors 7 (TLR7). TLR signaling induces maturation of dendritic cells and the production of a variety of inflammatory cytokines that are crucial for both innate and adaptive immunity. TLR7 is an intracellular receptor, which resides in endosomes and senses viruses to trigger host defence. Previous data have shown that TLR9 requires proteolysis to be functional but it is unclear whether other intracellular TLRs (TLR3 and TLR7) are also subject to degradation. Here, we used a protease deficient mouse model to show the *in vivo* importance of TLR7 processing in influenza infection. Inflammation monitored by cytokine release and adaptive immunity measured by cross priming of CD8^+^ T cells was significantly reduced in infected protease-deficient animals in comparison to control animals. We showed that TLR7 requires a proteolytic cleavage by a cysteine endopeptidase in order to be functional. Our findings indicate that TLR7 processing mediated by a protease, asparagynil endopeptidase, is critical for inducing robust anti-influenza immune responses. Given our results, targeting TLR7 response in the lungs through proteases may offer new therapeutic potential in pulmonary infection.

## Introduction

Influenza is a common respiratory disease where viral virulence can either cause just a moderate sickness or a severe pathology leading to hospitalisation or even death. There are studies demonstrating that IAV infection induces severe and aggressive innate response, manifested with excessive cytokine production by alveolar macrophages and respiratory epithelial cells [1], [2]. This innate immune response triggers the activation of professional antigen-presenting cells (APCs) leading to the initiation of adaptive immunity to eradicate the virus. Thus, CD8^+^ T cell priming to IAV requires antigen presentation by activated dendritic cells (DCs) that express co-stimulatory molecules and promote T cell differentiation and activation. Recent work has shown that tissue resident DCs from the lung are responsible for the presentation of exogenous antigens and subsequently the cross priming of T cells in a Toll like receptor 7 (TLR7)-dependent fashion [3], [4]. TLR7 senses single-stranded RNA from influenza viruses within the endosomes and has been shown to be essential in the induction of anti-viral immune responses to IAV [1], [5], [6], [7], [8].

Toll like receptors (TLRs) detect a wide variety of microbial products and in DCs they are crucial in linking innate to adaptive immunity [9]. TLRs contain several leucine rich repeats (LRR) in an extracellular loop, a trans-membrane domain and a cytosolic domain and are expressed either at the plasma membrane or in the endosomal/lysosomal organelles. TLR stimulation is linked to MyD88 or TRIF-dependent signaling pathways that regulate the activation of different transcription factors, such as NF-κB [10]. Specific interaction between TLRs and their ligands activates NF-κB resulting in enhanced inflammatory cytokine responses, induction of DC maturation and expression of chemokine receptors [11]. Little is known about how intracellular TLRs (TLR3, 7, 9) and their ligands are targeted to the endocytic pathway. Intracellular TLRs are sensitive to lysomotropic agents that neutralize acidic compartments such as chloroquine or concanamycin B indicating a role for endo/lysosomal proteases for their signaling. Indeed, recent findings have described the importance of proteolysis for TLR9 function [12], [13]. It has been shown that murine TLR9 is non functional until it is subjected to proteolytic cleavage in the endosomes. Upon stimulation, full-length (FL) TLR9 is cleaved into a C-terminal (C-ter) fragment sufficient for signaling. Many proteases, mainly cathepsins, have been shown to participate in this process in macrophages and in different cell lines [12], [13], [14]. However, in primary DCs, cathepsin K (CatK) and asparagine endopeptidase (AEP) are important in TLR9 processing. In CatK deficient DCs, TLR9 signaling was totally abrogated and in DCs lacking AEP, TLR9 cleavage in phagosomal compartments as well as CD4^+^ antigen specific T cell proliferation was greatly reduced upon CpG stimulation [15], [16]. Still, it is unclear whether TLR7 is also subject to proteolysis [12], [13], [17] and whether TLR7 processing is an important criterion for the immune response to influenza infection

Here, we describe for the first time that a protease, AEP, plays an important role in anti-influenza virus immune responses. Indeed, AEP-dependent TLR7 activation mediates inflammation, cross presentation of cell-associated antigens and cross priming of CD8^+^ T cells upon lung IAV infection. We demonstrate that AEP activity is critical for TLR7 processing *in vitro* and signaling *in vivo*. Altogether, these data indicate that endosomal processing is a prerequisite for intracellular TLR signaling and identify AEP as a major actor in anti-viral immunity.

## Results

### TLR7-dependent influenza virus inflammation and cross presentation of cell associated antigens require AEP

**Figure 1:**
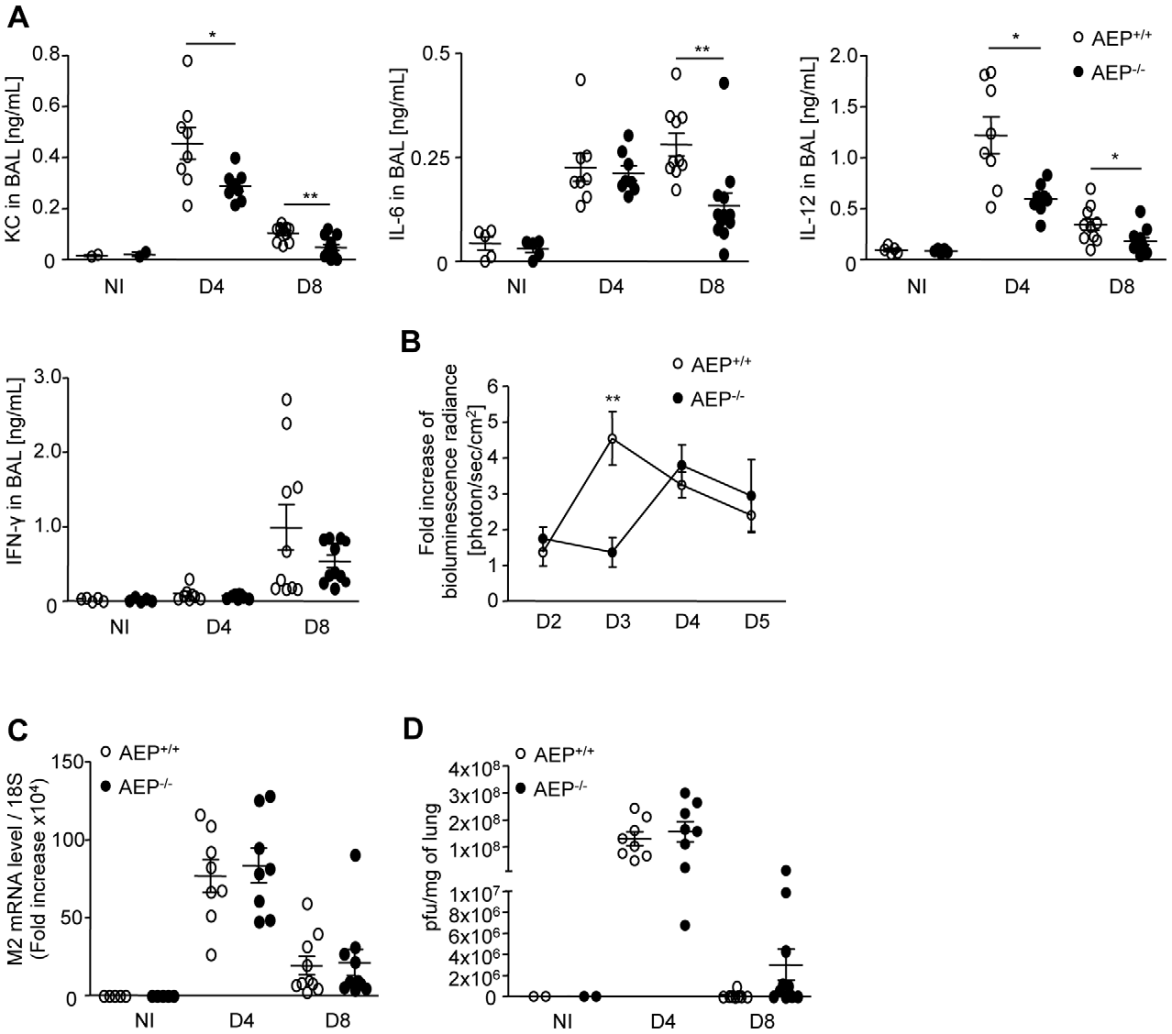
Reduced inflammation in IAV-infected AEP^−/−^ mice. (A) Broncho-alveolar lavage (BAL) fluid levels of KC, IL-6, IL-12 and IFN-γ in wt and AEP deficient mice before (NI), 4 d and 8 d after intranasal injection of IAV PR8 strain (100 pfu/mice). (n = 8 animals; graphs show mean ± SEM of two independent experiments, * p<0.05, ** p<0.01). (B) NF-κB activity in the lungs of WT or AEP deficient mice infected with IAV. Mice were co-infected at d 0 with IAV (100 pfu) and Ad-NF-κB-luc (2.5×10^8^,pfu). Bioluminescence was measured after instillation of 50 µl of luciferine (500 µg/mL) and photon emission was captured at different times post-infection using the IVIS system. Graph shows fold increase of NF-κB activity that represents the average radiance (photons/sec/cm^2^) of IAV+Ad-NF-κB-luc infected mice relatively to the average radiance corresponding to Ad-NF-κB-luc infected mice (n = 7–10 animals, mean ± SEM of three independent experiments, * p<0.05). (C–D) Viral titers in lungs of wt and AEP^−/−^ mice 4 d and 8 d post-viral challenge were determined by qRT-PCR performed on M2 viral protein (C) or by plaque assay (D). (pfu, plaque-forming units, n = 8 animals; graphs show mean ± SEM of two independent experiments).

To address the contribution of AEP in the innate immune response to influenza virus-infection, wild type (wt) and AEP deficient mice (AEP^−/−^) were infected with a sub-lethal dose of IAV (100 pfu) and the inflammation was monitored in the lungs at days 4 and 8 post-infection [18]. In AEP^−/−^ mice, significant inhibition of cytokine and chemokine secretion associated with influenza virus-induced pneumonia, including keratinocyte chemo-attractant (KC), interleukin 6 (IL-6), and interleukin 12 (IL-12), was detected compared to wt mice (Figure 1A). In addition, interferon gamma (IFN-γ) and interferon alpha (IFN-α) production was also reduced in AEP^−/−^ mice infected with IAV (Figure 1A and Figure S1A). No difference in IFN-β mRNA expression was detected between wt and AEP deficient mice (Figure S1B).

As NF-κB is the major pro-inflammatory transcriptional factor and regulates the induction of most inflammatory cytokines [19], we thus analyzed the contribution of the NF-κB pathway during the *in vivo* IAV infection. We used an adenovirus containing an NF-κB response element linked to a luciferase reporter gene (Ad-NF-κB-luc) [20]. Control WT or AEP^−/−^ mice infected with Ad-NF-κB-luc alone presented weak but similar bioluminescence scores (data not shown). Wt mice co-infected with the Ad-NF-κB-luc and IAV developed an important inflammatory reaction in lung tissue with a peak of NF-κB-activity at day 3, corresponding to the maximum of viral replication. In contrast AEP^−/−^ mice showed a moderate NF-κB activity (Figure 1B). Yet, the absence of AEP did not diminish the accumulation of innate inflammatory infiltrate characterized as macrophages/monocytes, granulocytes, and dendritic cells upon IAV administration (Figure S1C). Nonetheless, we noticed that the myeloperoxidase activity, which mirrored the neutrophil degranulation, was decreased in the broncho-alveolar lavage (BAL) of AEP^−/−^ mice compared to wt mice (Figure S1D). To ascertain whether the difference in inflammation we observed between wt and AEP^−/−^ mice was due to a deficit in viral clearance, viral titers of influenza were quantified either by mRNA expression of the M2 viral protein (Figure 1C) and by plaque assays (Figure 1D) in the lungs on days 4 and 8 post infection. No significant difference was detected between wt and AEP^−/−^ mice.

TLR7 has previously been shown to sense single-stranded RNA from viruses including IAV [7]. To confirm the role of TLR7 in the infectious model of IAV, we performed similar experiments in TLR7^−/−^ mice. We found reduced level of proinflammatory cytokines in the BAL of TLR7^−/−^ infected mice and to a similar level as in AEP^−/−^ infected mice in comparison to wt mice (Figure S2A).

**Figure 2:**
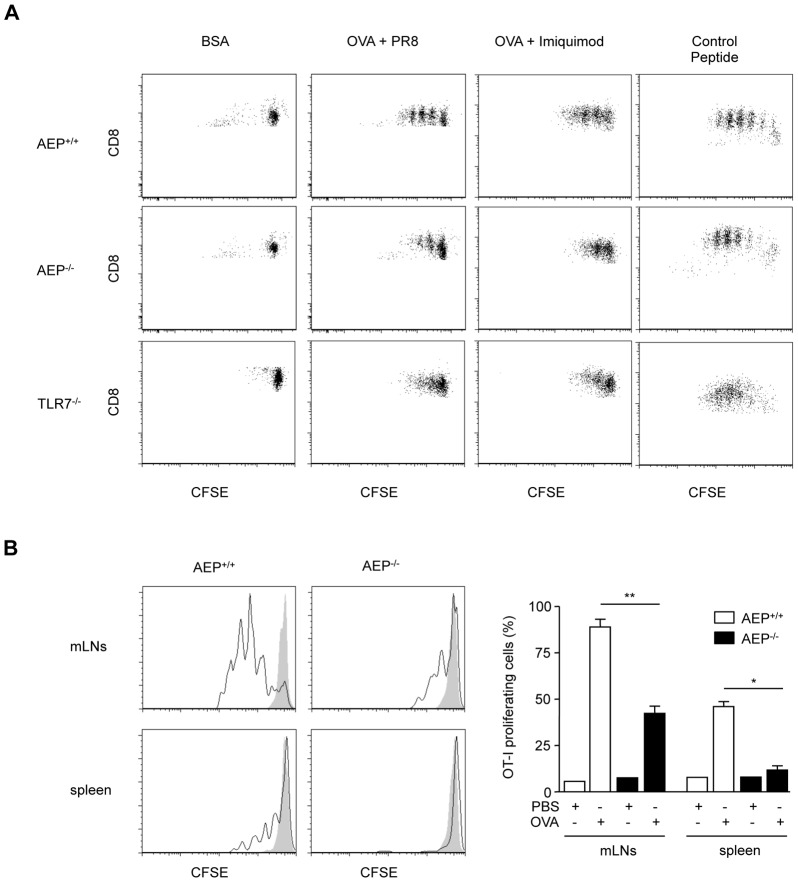
Impaired cross presentation in AEP deficient DCs and mice following IAV infection. (A) Proliferation of OT-I T cells cultured with DCs from wt, AEP^−/−^ or TLR7^−/−^ incubated with splenocytes from Balb/C mice (H-2^d^) electroporated with OVA or BSA and infected with PR8 virus or stimulated with imiquimod. SIINFEKL was used as an OVA-peptide control. Results are representative of three independent experiments. (B) Proliferation of OT-I T cells in the mLNs and the spleen 72 h after adoptive transfer of CFSE-labeled (CD45.1) OT-I T cells into IAV-infected wt or AEP^−/−^ mice (CD45.2) and intranasal treatment with PBS (gray histogram) or 120 µg of OVA (white histogram) 24 h later. Cells are gated on CD45.1 and histograms are representative of one mouse per group (left panel) and right panel represents the percentage of OT-I proliferating cells (n = 4 animals, mean ± SEM, * p<0.05, ** p<0.01).

Recently, studies have shown that cross presentation and cross priming of CD8^+^ T cells can be enhanced by signaling through TLRs expressed by DCs. Thus, we tested the hypothesis that reduced antigen specific T cell proliferation will be detected in AEP deficient cells and mice following IAV infection. We infected splenocytes from Balb/C mice (H-2^d^) electroporated or not with ovalbumin with the influenza virus PR8, and incubated them with wt, AEP^−/−^ or TLR7^−/−^ DCs together with T cells specific for ovalbumin (OT-I cells). T cell proliferation was analyzed 3 days later. To exclude that OVA processing was dependent on AEP, we incubated splenocytes expressing OVA alone with wt or AEP^−/−^ DCs together with OT-I cells. As shown in Figure S3, OT-I proliferation was comparable between wt and AEP^−/−^ cells. In wt cells, T cell stimulation was increased when DCs were incubated with IAV infected cells expressing OVA (Figure 2A, upper panel and Figure S3) but not with BSA-electroporated cells. In contrast, very weak proliferation of OT-I T cells was seen with AEP^−/−^ and TLR7^−/−^ DCs (Figure 2A, middle and lower panel). Similar results were obtained when a synthetic ligand of TLR7 (imiquimod) instead of IAV was used to trigger DCs activation (Figure 2A and Figure S2). In the three cell types (wt, AEP^−/−^, TLR7^−/−^) tested, OVA control peptide (SIINFEKL) triggered similar proliferation (Figure 2A). In addition, a synthetic ligand of TLR3 (poly(I∶C)) did also elicit proliferation of OT-I T cells but to the same extent in wt and AEP^−/−^ DCs (Figure S3).

To address *in vivo* whether antigens captured in the lungs could be cross-presented and prime CD8^+^ T cells in the mediastinal lymph nodes (mLNs) after influenza virus infection, we transferred CFSE labeled OT-I cells specific for ovalbumin into wt or AEP^−/−^ mice that had been previously infected with IAV. The mice were then challenged with ovalbumin or PBS intranasally and 3 days later proliferation of OT-I T cells in the draining lymph nodes (mLNs) and in the spleen was analyzed. We detected OT-I T cells proliferation in OVA-treated mice but not in PBS-treated mice (Figure 2B). T cells proliferation was stronger in OVA-treated wt mice whereas OT-I cells proliferate significantly less when injected in AEP^−/−^ (Figure 2B) and TLR7^−/−^ mice (data not shown). We conclude that AEP and TLR7 have a critical role in cross presentation, cross priming and cytokine secretion upon IAV infection.

### Reduced TLR7 signaling in dendritic cells and in mice deficient for AEP

**Figure 3:**
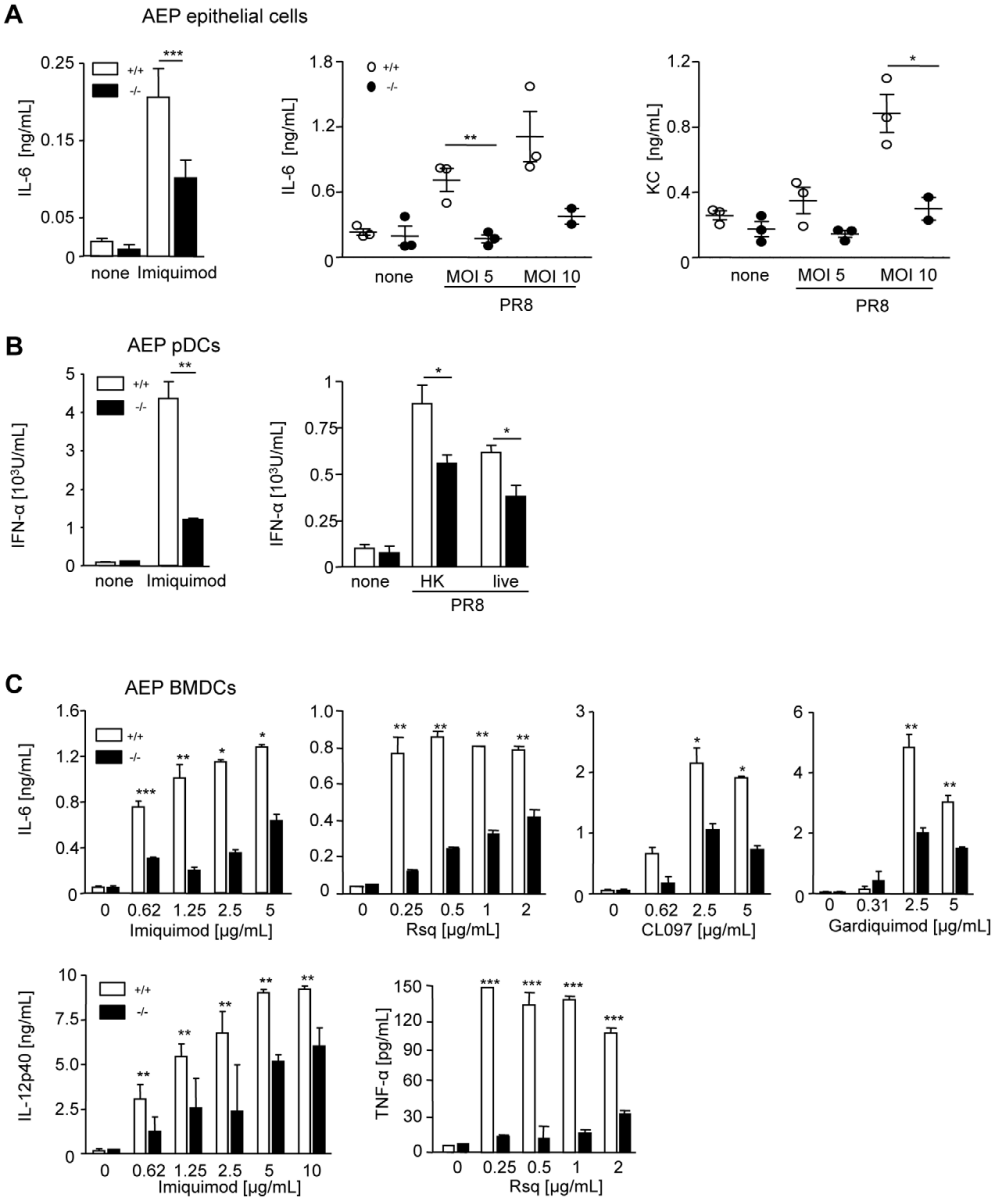
AEP activity is required for full cytokine production following TLR7 stimulation. (A, B) IL-6, KC or IFN-α secretions in supernatants of AEP^+/+^ (white bars) or AEP^−/−^ (black bars) lung primary epithelial cells (A) or pDCs activated (B) with 10 µg/mL of imiquimod or with the IAV virus PR8 heat killed (HK) or live at a multiplicity of infection = 1 or 5 for 16 h or 24 h. (n = 2–3; mean ± SEM, * p<0.05, ** p<0.01, *** p<0.001). (C) BMDCs from AEP^−/−^ mice (black bars) and from their wild type littermates (white bars) were stimulated with different TLR ligands for 16 h and secretion of IL-6, IL-12p40 and TNF-α in supernatants was measured by ELISA. (n = 2–6, mean ± SEM, * p<0.05, ** p<0.01, *** p<0.001).

The findings that both AEP^−/−^ and TLR7^−/−^ mice infected with IAV secreted less proinflammatory cytokines and had a defect in cross priming suggested that AEP might interfere with TLR7 signaling. To test this hypothesis, AEP^−/−^ and wt immune cells from myeloid and non-myeloid origins were generated and TLR7 response was monitored. Wt, TLR7 and AEP deficient lung epithelial cells, which are the first cells that will encounter IAV after infection, were purified and stimulated with imiquimod or IAV. Imiquimod response was totally abrogated in TLR7^−/−^ epithelial cells demonstrating the expression of TLR7 in these cells. In contrast, TLR7^−/−^ epithelial cells were still able to secrete cytokines following IAV infection probably because TLR7 also respond to IAV via RIG-I (Figure S2B). Yet, AEP^−/−^ lung epithelial cells showed a significant decrease in IL-6 and KC production upon imiquimod or IAV infection as compared to wt cells (Figure 3A). We further purified mouse plasmacytoid dendritic cells (pDCs), key cells involved in the secretion of IFN-α during viral infection. Upon imiquimod stimulation or IAV infection, mouse AEP^−/−^ pDCs showed a significant reduction in IFN-α production (Figure 3B). As expected, IAV stimulation was dependent on TLR7 (Figure S4A). To address the role of other proteases in TLR7 signaling, bone marrow derived DCs (BMDCs) from AEP^−/−^, CatB^−/−^, CatK^−/−^, CatL^−/−^ and CatS^−/−^ mice were generated and stimulated with TLR4 or TLR7 agonists. We observed a significant decrease in IL-6 secretion by BMDCs lacking AEP compared to wt cells upon intracellular TLR7 engagement (Figure 3C), but not when BMDCs deficient for CatB, CatK, CatL and CatS were stimulated (Figure S4B). Similar results were obtained in AEP^−/−^ DCs when IL-12p40 and tumor necrosis α (TNF-α) were measured or when other TLR7 agonists were used (Figure 3C). In addition, no differences in IL-6 production were detected between wt and cathepsin- or AEP-deficient cells when plasma membrane TLR4 was triggered (Figure S4C). AEP was shown to regulate cathepsin B, H, K and L maturation [16], [21], [22]. We have previously reported that the activity of these cysteine proteases is either not changed or increased at the steady state in the absence of AEP [16]. Nevertheless, to be sure that the diminution in TLR7 signaling we detected in AEP^−/−^ DCs was not influenced by reduced cathepsin activity, we monitored cathepsins B, K, L and S activities in the presence of imiquimod in wt and AEP^−/−^ cells. Using fluorogenic substrates selective for CatB, CatB and CatL, CatK or CatS, we found an increase in the activity of these cysteine proteases in AEP^−/−^ DCs in comparison to wt cell (Figure S4D). These data indicate that impaired TLR7 signaling in AEP deficient DCs is not associated with reduced activity of other cathepsins. In contrast, in cathepsin K deficient DCs, AEP activity was decreased (Figure S4E) which might explain the weak but not significant reduction of TLR7 signaling detected in cells lacking CatK. Together these results demonstrate that cells from different origins lacking AEP have diminished cytokine secretion upon TLR7 activation and IAV infection.

**Figure 4:**
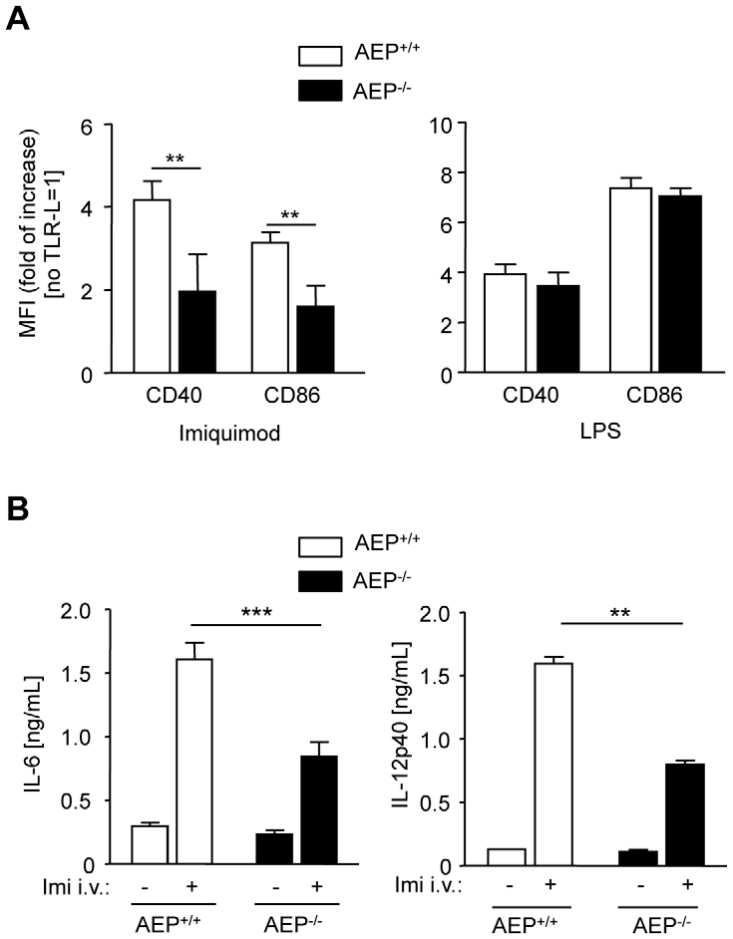
Decreased cytokine and co-stimulatory molecule expression in AEP^−/−^ DCs *in vivo* upon TLR7 ligand sensing. (A) FACS analysis of *in vivo* maturation of spleen CD11c^+^ cells 4 h after i.v. injection of 10 µg of imiquimod (left panel) or 1 µg of LPS (right panel) compared to no TLR ligand stimulation equivalent to 1. (B) IL-6 (left panel) and IL-12p40 (right panel) secretion were measured in serum of AEP^+/+^ or AEP^−/−^ mice 2 h after i.v. injection with imiquimod or PBS. (n = 9 animals for imiquimod; n = 2 animals for PBS; mean ± SEM for A and B, ** p<0.01, *** p<0.001).

To investigate the role AEP plays in TLR7-stimulation of DCs *in vivo*, we next assessed the ability of AEP-deficient DCs to mature phenotypically and to secrete pro-inflammatory cytokines. It was previously reported that DCs are the main target of TLR stimulation after two hours of TLR ligands injection in mice [23]. DC maturation in the spleen was examined after intravenous injection with imiquimod or LPS. In control mice, both LPS and imiquimod induced an increased expression of CD40 and CD86 in the CD11c^+^ spleen cells. These responses were compromised in AEP^−/−^ mice injected with imiquimod (Figure 4A). In addition, we consistently observed attenuated IL-6 and IL-12p40 secretion in the serum of AEP^−/−^ mice following TLR7 stimulation as compared to wt mice (Figure 4B). To further investigate that the decrease in TLR7 signaling observed *in vivo* was specific to AEP, we performed the same experiments in CatB deficient mice as we have noticed a little reduction in TLR7 stimulation in BMDCs lacking CatB (Figure S4B). We noticed similar or even increase expression of costimulatory molecules in spleen CD11c^+^ cells purified from CatB^−/−^ mice and similar IL-6 secretion in the serum of CatB^−/−^ mice, as compared to wt mice (Figures S5A and S5B). We conclude that, *in vivo*, AEP is required for the secretion of inflammatory cytokines and for the phenotypic maturation of CD11c^+^ cells induced by TLR7 engagement.

### AEP generates a TLR7 C-ter fragment

**Figure 5:**
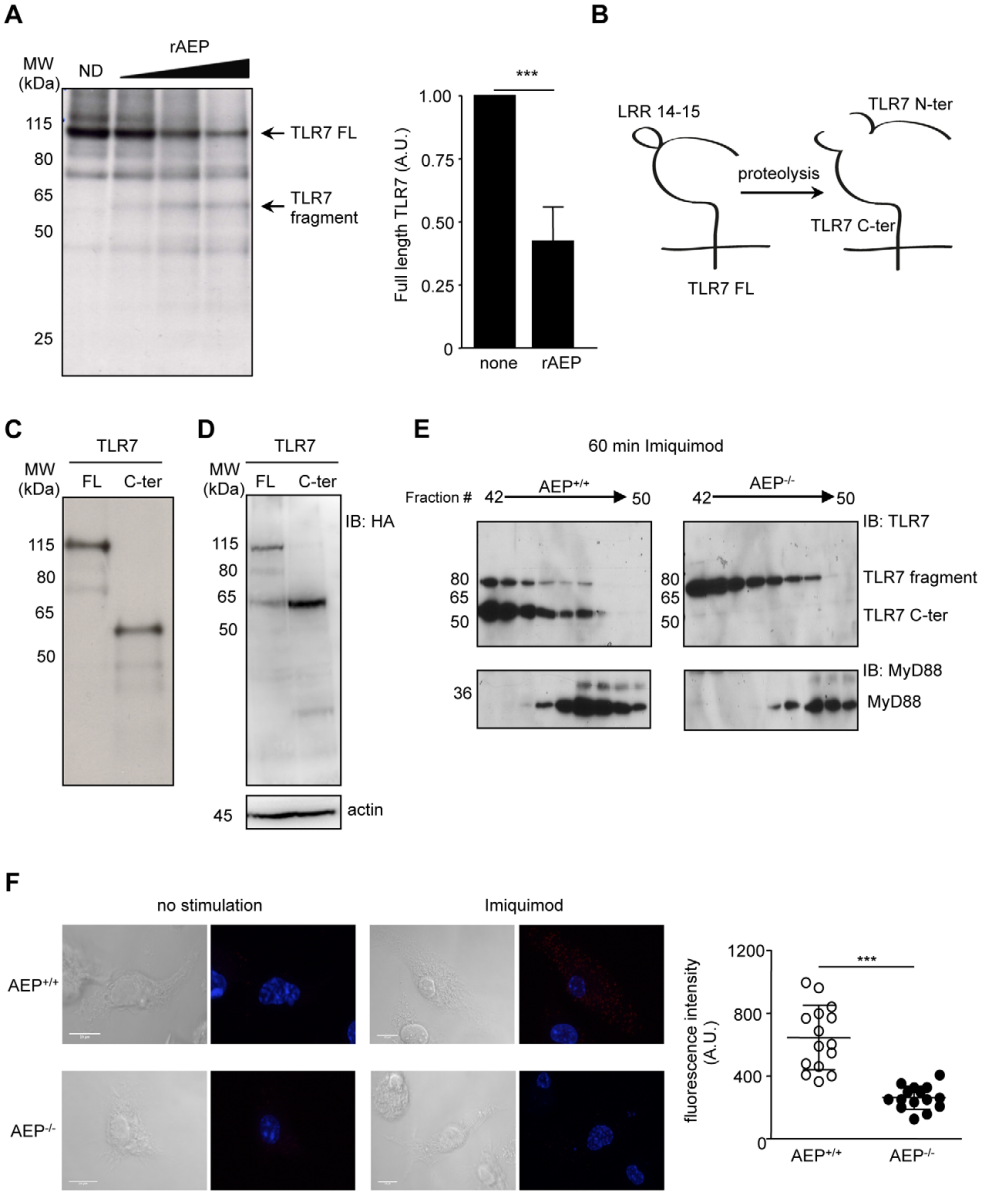
AEP generates a TLR7 C-ter fragment *in vitro* and in primary DCs. (A) *In vitro* transcription and translation of mTLR7 FL followed by 2 h digestion of radiolabelled mTLR7 FL with different doses of rAEP (3.25 to 15U) (left panel). ImageJ software quantification of TLR7 digestion with 15U rAEP (right panel). Mean of three different experiments normalized to undigested TLR7. *** p<0.001 (ND: non-digested). (B) Schematic representation of TLR7 protein and its cleavage fragments. (C) *In vitro* transcription and translation of mTLR7 FL and C-ter cDNAs labeled with S^35^. (D) Fibroblast were transfected with cDNAs encoding for HA-tagged TLR7 FL or C-ter together with UNC93B1. 48 h later, cells were stimulated with imiquimod for 1 h and western blot was performed with an anti-HA antibody. (E) Anti-TLR7 and MyD88 immunoblot of HPLC fractions of lysates from AEP^+/+^ (left panel) or AEP^−/−^ BMDCs (right panel) treated with imiquimod (5 µg/mL) for 60 min. (C–E) Representative data from three independent experiments. (F) Detection of MyD88 and TLR7 interaction using DuoLink *in situ* with anti-MyD88 and anti-TLR7 specific mAbs on AEP^+/+^ or AEP^−/−^ BMDCs stimulated or not with imiquimod for 30 min. PLA signals are shown in green and the nuclei in blue. Quantification of mean fluorescence using Image J software (n = 11), *** p<0.001.

We next addressed whether AEP could process TLR7 *in vitro*. Radiolabelled mouse TLR7 was incubated with different recombinant cysteine (cathepsins B, K, L, S) and aspartic proteases (cathepsin D) harboring the same enzymatic activity. Upon incubation of TLR7 with different doses of recombinant AEP, TLR7 FL was degraded to produce one major band of 60 kDa size (Figure 5A). *In vitro* translated TLR7 digested with a higher concentration of AEP showed two distinct bands migrating approximately around 60 and 30 kDa (TLR7 fragments, Figure S6A). As shown in Figure S6A, cathepsin L (CatL) and cathepsin S (CatS) were also able to degrade totally full-length (FL) TLR7. However, when decreasing CatL and CatS concentrations were used to digest FL TLR7, no defined processing products were detected (data not shown). *In vitro* digestion assays using human TLR7 also showed processing of full-length TLR7 by AEP and cathepsins L and S (Figure S6B). To verify whether the fragment generated upon AEP digestion had the same size as a putative C-terminal (C-ter) fragment, we predicted a candidate cleavage site in TLR7 by homology with TLR9 [12] (Figure 5B) and translated this C-ter cDNA *in vitro* (Figure 5C). Indeed, the putative candidate TLR7 C-ter fragment migrated approximately at the same size as the TLR7 product observed after TLR7 digestion by AEP (Figures 5A and 5C). We next assessed if the TLR7 C-ter fragment was produced in cells. We transfected fibroblasts with cDNAs encoding HA-tagged full length or HA-tagged C-terminal TLR7 and 48 hours later, transfected cells were lysed and blotted with an antibody directed against HA. In cells stimulated with imiquimod for 1 h, TLR7 full-length was detected as well as two fragments migrating around 80 kDa and 60 kDa (Figure 5D). The 60 kDa processing fragment, which appeared upon imiquimod incubation, migrated at the same size as the TLR7 C-ter we designed *in vitro* (Figures 5C and 5D). In addition, to assess whether the TLR7 C-ter fragment was produced in primary DCs, we purified phagosomes from wt and AEP^−/−^ cells fed with magnetic particles. At the steady state, TLR7 FL and C-ter were observed in early (20 minutes) and late (120 minutes) phagosomes. In the presence of imiquimod, TLR7 cleavage occurs more rapidly and was already detectable after 20 min (Figure S7A).

To investigate whether the TLR7 C-ter fragment is generated in AEP^−/−^ cells and recruits the adaptor molecule MyD88, we stimulated wt or AEP^−/−^ BMDCs with imiquimod and fractionated TLR7 and MyD88 on the basis of size exclusion chromatography as we previously did for TLR9 [16]. Similar chromatography profiles were obtained for both cell types (data not shown). After 60 min of imiquimod stimulation in wt cells, two TLR7 products migrating around 80 kDa and 60 kDa (Figure 5E, left panel) were detected. As before, the 60 kDa product had a similar size as the TLR7 C-ter fragment we observed in the *in vitro* translation assay (Figures 5C and 5D). In the absence of AEP, no TLR7 processing fragment of 60 kDa was detected (Figure 5E, right panel). We then monitored if MyD88 co-eluted with the C-ter form of TLR7. Indeed, in wt cells we could detect MyD88 in fraction 47 where TLR7 C-ter product was present (Figure 5E, left panel). To look at MyD88-TLR7 interaction, we used the DuoLink method that gives a signal if two different antibodies are localized within 40 nm of each other. This method allows us to detect and visualize TLR7 conjugated to MyD88. We could confirm association between MyD88 and TLR7 in wt cells only when the cells were stimulated with imiquimod (Figure 5F, upper panel). In AEP^−/−^ cells, significatively less signal was detected (Figure 5F, lower panel).

In conclusion, AEP generates a TLR7 processing fragment, which is produced in wt cells upon imiquimod stimulation but not in AEP^−/−^ DCs. In addition, a reduced association between cleaved TLR7 and the adaptor protein MyD88 is observed in AEP^−/−^ cells.

### TLR7 C-terminal fragment is competent for signaling and is dependent on acidic pH

**Figure 6:**
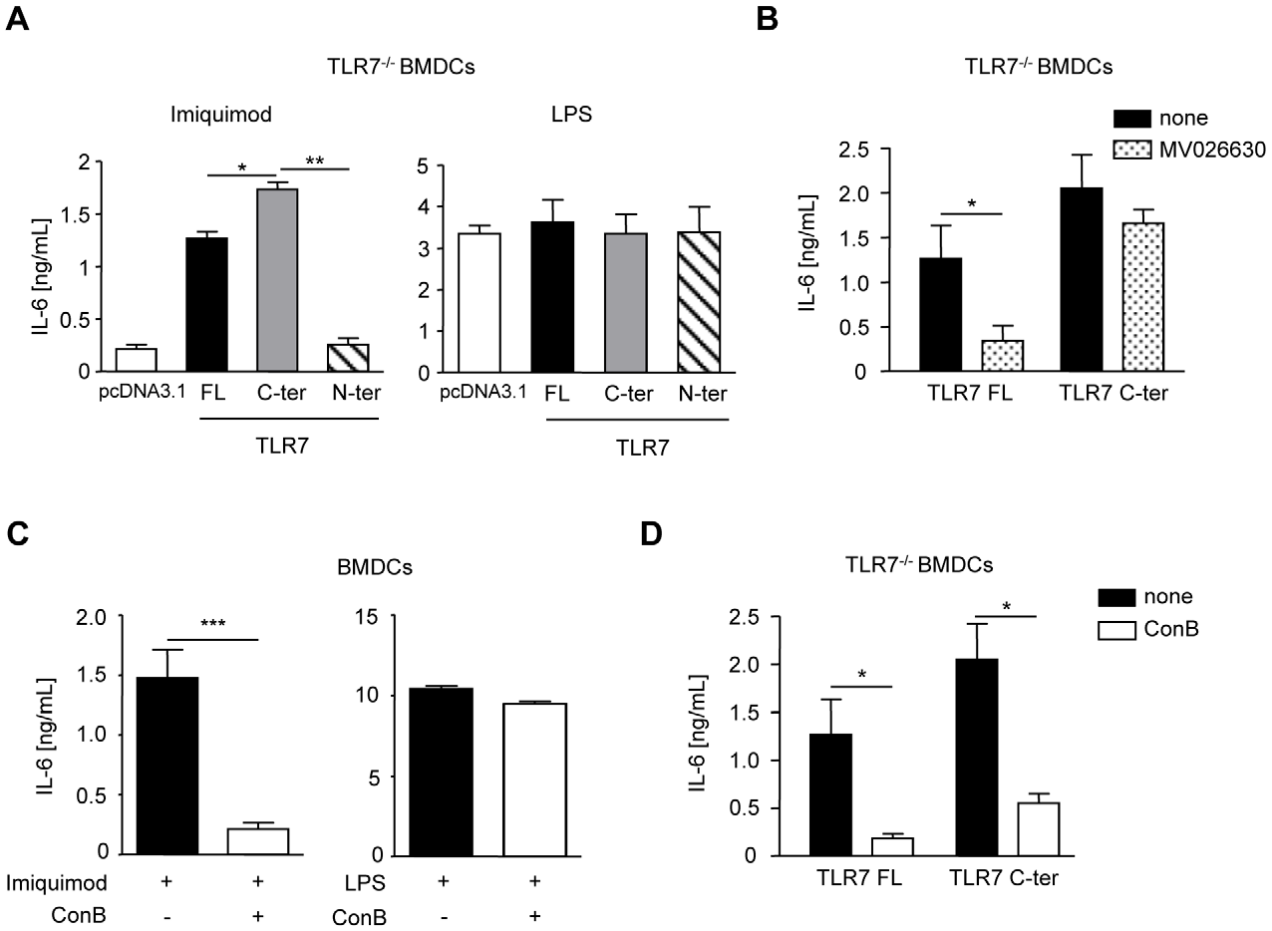
TLR7 C-ter is functional and requires acidic environment. (A) IL-6 secretion in TLR7^−/−^ BMDCs transfected with pcDNA3.1, FL, C-ter or N-ter TLR7 cDNAs and stimulated with 5 µg/mL of imiquimod or 10 ng/mL of LPS for 16 h. (n = 3–4; mean ± SEM, * p<0.05, ** p<0.01). (B) IL-6 secretion in TLR7^−/−^ BMDCs transfected with FL or C-ter TLR7 and stimulated with imiquimod as above and treated with or without 25 µM MV026630 (AEP inhibitor). (n = 3; mean ± SEM, * p<0.05). (C) IL-6 secretion in BMDCs activated with either 5 µg/mL imiquimod or 10 ng/mL LPS for 16 h, in the presence or not of 20 nM of concanamycin B. (n = 3; mean ± SEM, ** p<0.01, *** p<0.001). (D) IL-6 secretion in TLR7^−/−^ BMDCs transfected with FL or C-ter TLR7 and stimulated with imiquimod and treated as above. (n = 3; mean ± SEM, * p<0.05).

We next assessed whether the TLR7 C-ter fragment is functional. TLR9 C-ter fragment containing a part of the ecto-, trans-membrane and cytoplasmic domains has been shown to be fully competent for signaling at a similar level as FL TLR9 [12]. We next wondered whether the same was true for TLR7. Indeed, the C-terminal fragment of TLR7 was sufficient to initiate signaling and even to a higher level than full-length TLR7 when expressed in TLR7^−/−^ DCs upon different doses of imiquimod stimulation (Figures 6A, left panel, S7B and S7C). Neither the empty vector nor the TLR7 N-ter fragment could restore signaling in DCs lacking TLR7 (Figure 6A, left panel). As expected, LPS-stimulated cells transfected with the same constructs secreted similar amount of IL-6 (Figure 6A, right panel). In addition, cells pretreated with the specific AEP inhibitor (MV026630) failed to restore cytokine secretion in FL TLR7-transfected TLR7^−/−^ DCs (Figure 6B). On the contrary, TLR7 C-terminal fragment did not require any further processing because its signaling was not reduced when AEP was inhibited (Figure 6B).

We next tested whether the C-terminal fragment of TLR7 could restore full TLR7 signaling in DCs lacking AEP. AEP^−/−^ cells were transfected with the cDNAs encoding TLR7 full-length vector or the recombinant C-terminal TLR7 product. After TLR7 stimulation, diminished IL-6 secretion, as expected, was detected when DCs lacking AEP were transfected with the cDNA encoding full length TLR7 as compared to wt cells (Figure S8A, left panel). On the contrary, TLR7 C-terminal fragment restored signaling in AEP^−/−^ cells to the same level as in wt cells after imiquimod stimulation as indicated by IL-6 secretion (Figure S8A, left panel). As a control, LPS-stimulated cells transfected either with full-length or C-terminal TLR7 plasmids produced same amount of IL-6 (Figure S8A, right panel). However, we noticed that the difference in TLR7 response between wt and AEP^−/−^ DCs transfected with the cDNA encoding for the FL TLR7 was not as significant as when AEP^−/−^ DCs were stimulated with imiquimod. We suspect this might be due to the overexpression of the TLR7 protein in these experiments. In summary, our results suggest that TLR7 processing fragment is sufficient and necessary for TLR7 activity.

AEP is a cysteine protease that requires acidic pH, like most of the other cysteine and aspartic proteases, for its optimum activity [24]. We and others have previously shown that the endosomal pH of DCs is rather neutral and decreases only in lysosomal compartments [16], [25]. We then decided to monitor the pH in endosomes (where most likely TLR7 traffics) and lysosomes of DCs upon imiquimod stimulation using specific probes [25], [26]. A drastic drop of endosomal pH was detected upon imiquimod stimulation, which persisted upon time and was also detected in lysosomes, in comparison to unstimulated cells (Figure S8B). The drop of pH upon imiquimod stimulation was also observed in AEP deficient DCs (Figure S6B). This increase in endosomal/lysosomal acidification correlates with a boost in AEP activity measured in total cell lysate (Figure S8C). This enhancement of AEP activity (about 2 fold) was particularly striking in phagosomes where most likely cleavage of TLR7 occurred after imiquimod stimulation (Figure S8D). Cathepsins B, K, L and S activities were also increased upon TLR7 stimulation (Figure S8E) indicating that it was not specific for AEP. To verify the importance of endosomal acidification in TLR7 signaling, we treated cells with concanamycin B (a drug interfering with pH acidification by blocking the recruitment of the Vo subunit of the V-ATPase complex) and measured IL-6 secretion after imiquimod stimulation. Treatment with concanamycin B completely abrogated TLR7 signaling but not TLR4 response as expected (Figure 6C). Furthermore, endosomal acidic pH was still required for the TLR7 C-ter product to signal suggesting that optimal ligand receptor binding might occur at low pH [27] (Figure 6D) and/or acidification might be required for a change of conformation allowing the TLR7 C-ter fragment to bind the adaptor molecule MyD88 and to signal. Thus, TLR7 processing requires low pH and AEP activity.

### Mutation of AEP cleavage site between LLR 14–15 abrogates TLR7 signaling

**Figure 7:**
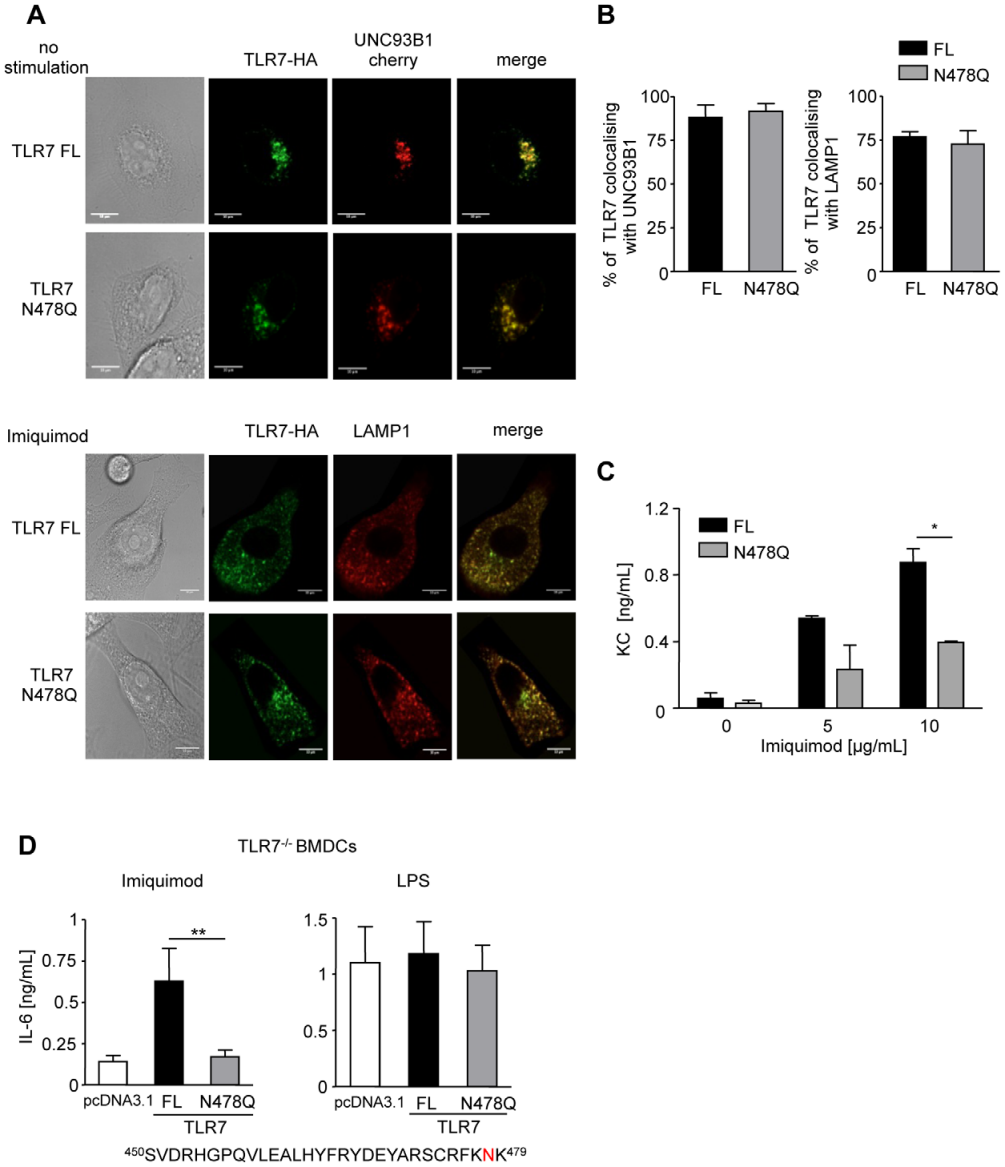
Mutating a putative AEP cleavage site abrogates TLR7 signaling. (A) Immunofluorescence microscopy of resting (top) or imiquimod activated (bottom) fibroblast co-transfected with FL or N478Q TLR7 HA-tagged and UNC93B1-cherry cDNAs and stained for TLR7 (green), LAMP1 (red) and UNC93B1 (red). One representative experiment out of three shown. (B) Quantification of colocalization using Image J software (n = 8). (C) KC secretion in fibroblasts transfected with FL or N478Q TLR7and stimulated overnight with 10 µg/mL imiquimod. (n = 2; mean ± SEM, * p<0.05). (D) IL-6 secretion in TLR7^−/−^ BMDCs transfected with FL or N478Q TLR7 and stimulated with TLR ligands as above. (n = 4; mean ± SEM, ** p<0.01).

AEP cleaves native antigens at asparagine sites [28]. So, it is tempting to speculate that AEP cleaves directly TLR7. Indeed, the residues 450–479 in TLR7 situated between the two leucine rich-regions (LRR 14–15) described to be susceptible for proteolysis and to be a target for AEP cleavage in TLR9 [12], [13], [16], contain also an asparagine that can be a putative cleavage site for AEP. To test this hypothesis, we mutated asparagine residue 478 to glutamine. We first investigated the intracellular localization of this mutant and wt TLR7 when expressed in transfected in fibroblasts. To allow proper TLR trafficking, wt or mutated TLR7 cDNAs were transfected together with a cDNA encoding for the chaperone molecule, UNC93B1 [29]. At the steady state, both wt and mutated TLR7 reside in the ER (Figure 7A, upper panel). Indeed, we could observe colocalisation between UNC93B1 (an ER marker) and both TLR7 constructs (Figure 7B, left panel). Upon imiquimod stimulation, wt and mutated TLR7 traffic to LAMP positive lysosomal compartments (Figure 7A, lower panel). No difference in intracellular localization was observed between TLR7 wt and the N478Q mutant suggesting that trafficking of TLR7 mutant is not impaired (Figure 7B, right panel). We then tested whether this mutant was able to signal following imiquimod stimulation when expressed in fibroblasts and TLR7^−/−^ DCs. We observed 70% to 90% of decrease in IL-6, KC and IL-12p40 secretion when TLR7 mutant was activated with imiquimod but not with LPS compared to wt TLR7 (Figures 7C, 7D and S7D). Transfection efficiency assessed by cytometry was the same for both constructs (20% in DCs and 90% in fibroblasts). Altogether these results suggest a direct cleavage of TLR7 by AEP generating a C-terminal fragment fully competent for signaling.

## Discussion

Influenza A virus is the agent of one of the most common infectious diseases and remains a major public health burden. Numerous studies have shown that the main cause of death after IAV infection is due to a “cytokine storm” or excessive secretion of proinflammatory cytokines [1]. Indeed, pharmacological intervention in order to limit cytokine responses and thus to reduce the immunopathogenicity of influenza virus has been shown to be beneficial for the host [1], [30], [31]. Signaling pathways in host IAV infected cells can be induced by a variety of viral components, among them double- and single-stranded RNA which are sensed by the cytosolic RNA helicases, RIG-I, and the TLR family members TLR7 and TLR3 [6], [32], [33], [34]. So far, the therapeutic approaches have targeted these last receptors.

Based on our previous results showing the critical role of AEP in the processing and signaling of another intracellular TLR, TLR9, it was tempting to speculate that AEP might be involved in TLR7 activation upon IAV infection. Interestingly, mice lacking AEP were unable to generate a strong antiviral immune response when challenged with the influenza virus strain PR8 described previously to be recognized by TLR7 [5], [6], [7], [8]. This was not due to a deficit in viral clearance because the viral titers of IAV remain the same in wt and AEP deficient mice. By contrast, lung epithelial cells, that provide the first spatial barrier against this respiratory virus, from AEP deficient mice responded much less to that viral challenge. Moreover, plasmacytoid dendritic cells that lack AEP expressed reduced amount of IFN-α compared to wt cells, demonstrating that the response of the very first sentinels upon viral infection is reduced in the absence of AEP. Besides this attenuated innate immune response, we were able to observe that the adaptive immune response in AEP deficient mice was also severely impaired. CD8^+^ T cell priming to IAV requires antigen presentation by activated DCs that express costimulatory molecules, which in turn promote T cell-differentiation and activation. We demonstrate that in AEP deficient mice the ability to initiate an immunogenic CD8^+^ T cell response to an exogenous antigen that is not synthetized by the antigen presenting cells was severely compromised. We ruled out the contribution of TLR3 [18] in our work because TLR3 signaling does not require AEP (data not shown) and we did not observe difference between wt and AEP^−/−^ DCs in OVA cross presentation when TLR3 was stimulated (Figure S3). Also, we cannot exclude the importance of the inflammasome in IAV infection [33], [35]. In our model, no difference in IL-1β secretion was detected between wild type and AEP deficient mice when the IAV PR8 strain was administered, suggesting that AEP does not play a role in the activation of the inflammasome (data not shown).

Recent results have shown that TLR7 is also subject to proteolytic degradation [17] but the proteases involved in this process have not been identified and the functionality of this product has not been determined. Indeed, no difference was observed between control DCs and DCs lacking different single cathepsins (Cathepsins B, K, L and S) in cytokines production following TLR7 stimulation [12], [13]. Furthermore, using Z-FA-fmk, a broad inhibitor of cysteine proteases and caspases, in a macrophage cell line, partial inhibition (about 30%) in TLR7 response was reported [17]. This altered signaling was not observed when AEP was blocked. We have not tested macrophage cell lines. However, using primary alveolar macrophages, we have not observed any difference in cytokine secretion between AEP-deficient macrophages and control cells following TLR7 stimulation. Indeed, we and others have shown that DCs behave differently from macrophages because they have evolved distinctly to regulate endosomal acidification at the steady state and upon TLR stimulation [16], [25], [36], [37]. The experiments presented in this paper establish that TLR7 processing is dependent on AEP in DCs and epithelial cells. The *in vitro* cleavage of TLR7 by AEP, the impaired cytokine secretion in DCs and in mice deficient for AEP following TLR7 stimulation or in DCs expressing a TLR7 mutant lacking the AEP cleavage site at position 478 support this conclusion. Interestingly enough, both TLR7- and TLR9-processing and -signaling depend on AEP. These two intracellular TLRs necessitate UNC93B1 for their trafficking [29], [38], share sequence homology [39], have similar expression in certain DC subtypes and both contribute to the development of some autoimmune diseases like systemic lupus erythematous [40].

In conclusion, AEP plays a major role in TLR7 signaling in primary epithelial and dendritic cells and contributes to the induction of inflammation and CD8^+^ T cell activation during influenza virus infection (Figure S9). Given our findings, targeting TLR7 response in the lungs through AEP may offer new therapeutic potential in pulmonary infection. Indeed, a specific inhibitor of AEP (MV026630) is available and could be tested in such inflammatory diseases.

## Material and Methods

### Ethics statement

Animals were housed in the Institut Pasteur animal facilities accredited by the French Ministry of Agriculture to perform experiments on live mice in appliance of the French and European regulations on care and protection of the Laboratory Animals (EC Directive 86/609, French Law 2001-486 issued on June 6, 2001). Protocols were approved by the veterinary staff of the Institut Pasteur animal facility (permit number 99.174) and were performed in compliance with the NIH Animal Welfare Insurance A5476-01 issued on 02/07/2007.

### Mice

AEP^−/−^ mice were generated in C. Peters' lab (Freiburg) and backcrossed 11 times on B6 background. Animals were bred in a pathogen-free environment in accordance with the Institut Curie guidelines. Cathepsin B, L, S deficient mice were a kind gift from A.M. Lennon (Paris, France) and mice lacking cathepsin K were kindly donated by P. Saftig (Germany).

### Influenza virus

Influenza A/Puerto Rico/8 [mouse adapted] P2.3 (H1N1, PR8) virus was generously provided by N. Naffakh (CNRS URA 3014, Institut Pasteur, Paris, France), was prepared as previously described [18] and the titer was expressed as plaque forming units (pfu)/mL [41].

### Cells and stimulations

Fibroblasts were obtained from the skin of C57Bl/6J mice following the protocol as previously described [42]. Bone marrow derived DCs were generated from AEP^−/−^, CatB^−/−^, CatK^−/−^, CatL^−/−^, CatS^−/−^, TLR7^−/−^ mice and their wt littermates as previously described [16]. Cell differentiation was controlled by FACS (anti-CD11c, HL3, and anti-CD11b, M1/70, BD biosciences). Splenic pDCs were isolated using mPDCA-1 selection kit (Miltenyi Biotech, 90% of purity as determined by FACS). Epithelial cells were isolated from the mouse lung as previously described [43]. Plated cells in 96-well plate were treated overnight with the TLR ligand (LPS (Sigma-Aldrich), Imiquimod, poly(I∶C), Resiquimod, or Gardiquimod, CL097 (Invivogen)). In some experiments BM-DCs were pre-incubated with ConB (20 nM, Sigma) for 20 min or for 1 h with the AEP specific inhibitor: MV026630 (25 µM, laboratory of H. Overkleeft) before adding the TLR agonist. Cytokines were measured in supernatants using home-made (IFN-α) or commercial (TNF-α, IL-6, IL-12p40, KC, IFN-γ, RANTES, eBioscience or R&D Systems) ELISA.

### *In vivo* IAV model

Male mice were anesthetized by ketamine-xylazine and infected intranasally with 50 µl of PBS containing 100 pfu of PR8 virus. Airways were washed 4×0.5 mL of PBS, and the BAL was collected to further determine cell differential counts (Beckman Coulter) and FACS. Different cytokines were measured in BALs. IFN-β protein expression was quantified by quantitative real-time RT-PCR in total RNA extracted from lung tissue using the RNeasy Mini Kit (Qiagen). Primer sequences were for murine *IFN-β* gene F: 5′CACAGCCCTCTCCATCAACT-3′ and R: 5′-TCCCACGTCAATCTTTCCTC-3′. In other experiments, recombinant adenovirus encoding for NF-κB-luciferase (Ad-NF-κB-luc, Gene Transfer VectorCore, University of Iowa) was intranasally co-injected (2.5×10^8^ pfu) with PR8 virus (100 pfu) in order to analyze NF-κB activation in the lungs. Photon emission of the luminescent construct transduced in the lungs was quantified using the IVIS system (Xenogen Biosciences) after intranasal injection of luciferine (50 µl, 500 µg/mL) as previously described [20].

### FACS analysis

BAL cells were stained with Live/Dead fixable near-IR dead cell stain kit (Invitrogen) in PBS for live/dead discrimination. Cells were then incubated with anti-CD16/32 in PBS-1% BSA and stained with PerCP-Cy5.5-anti-CD11b (clone M1/70), APC-anti-CD11c (clone HL3), PE-Cy7-anti-Gr-1 (Ly-6G and Ly-6C, clone RB6-8C5) (BD Biosciences). Cells were fixed with paraformaldehyde 2% and analyzed using a CYAN ADP cytometer (Beckman Coulter) and with FlowJo software.

### Quantification of M2 viral protein and particles

Total RNA was extracted from lung tissue using the RNeasy Mini Kit (Qiagen) and the M2 viral protein quantification performed by quantitative real-time RT-PCR as previously described [20]. Total proteins were extracted from lung tissue in PBS in order to quantify the remained IAV by determining pfu present in lung lysates with the method previously described [41].

### *In vitro*, *in vivo* cross-presentation and *In vivo* DCs stimulation

*In vitro* cross-presentation assays were performed as previously described [44]. Briefly, splenocytes from Balb/C mice (H-2^d^) were electroporated with 0.5 mg OVA or BSA and infected with PR8 virus (10 pfu) or stimulated with 10 µg/mL imiquimod or 100 µg/mL poly(I∶C) before irradiation (10 Gy) and incubation with DCs. Then, CFSE labeled OT-I T cells were added to the culture and the proliferation of T cells was monitored 3 days later. DCs incubated with SIINFEKL (100 ng/mL) were used as a proliferation control. *In vivo* cross-presentation assays were performed as previously described [4]. Briefly, CFSE labeled CD8^+^ T cells from OT-I RAG (CD45.1^+^) mice were intravenously injected at day 4 into IAV-infected CD45.2^+^ recipient mice, a day later OVA or PBS were administered intranasally and mice were sacrificed at day 8 to monitor proliferation of T cells in the mLNs. Mice were injected intravenously with TLR agonist or PBS alone and bled 2 h later. Serum cytokine was assessed by ELISA and splenic DCs were stained with anti-CD11c-APC (HL3), anti-CD86-PE (GL1) or anti-CD40-PE (3/23) (BD Biosciences) for flow cytometry analysis.

### Size chromatography exclusion, immunodetection and protease activity

50×10^6^ BM-DCs were stimulated with Imiquimod (10 µg/mL) for 60 min. Cells were then lysed in a buffer (50 mM Tris pH 7.4, 150 mM NaCl, 5 mM MgCl_2_) with 0.5% NP40 for 30 min on ice and centrifuged at 90000 rpm. The extract was applied to a G-200 Sephadex size exclusion column (Pharmacia). Fractions were eluted using the previously buffer and 5 µg of protein were immunoblotted for TLR7 (Imgenex) and MyD88 (Abcam) expression. Protease activities were assayed on a Mithras LB940 (Berthold technologies) by measuring the release of fluorescent N-Acetyl-Methyl-Coumarin in citrate buffer (pH 5.5) at 37°C as previously described [16].

### Endosomal pH measurement

Endosomal pH measurement has been described [16]. Briefly, cells were pulsed with 1 mg/mL of FITC- and Alexa-647-labelled 40 kDa dextrans (Molecular Probes) for 10 min at 37°C and washed with cold PBS-1% BSA. Cells that have endocytosed the probes were analyzed by FACS at different times.

### Phagosome preparation

50×10^6^ BMDCs were pulsed for 20 min at 37°C with magnetic particles (Invitrogen) and then chased at 37°C for 100 min in the presence or absence of imiquimod (10 µg/mL). Cells were then washed with PBS and mechanically disrupted by passing them through 25 g needle. Phagosomes were purified by magnetic separation, washed, and lysed. Equal amounts of proteins were submitted to separation on a 4–12% SDS NuPAGE Bis-Tris gels (Invitrogen). Proteins were transferred on a PVDF membrane and TLR7 immunodetection was realized with rabbit pAb anti-TLR7 (Imgenex).

### Constructs

Murine TLR7 constructs (described in Table 1) containing either the FL sequence, the C-terminal (aa 480–1052) or the N-terminal part (aa 1–449) followed by a HA-tag were cloned into pcDNA3.1 by PCR of the pUNO mTLR7-HA plasmid (Invivogen). The full-length mTLR7 construct was mutagenized in its Asn 478 into Gln using the Quick change mutagenesis kit (Stratagene).

**Table 1:** Sequences of primers used to TLR7 constructs.

Primer name	Sequence
**TLR7 Not I forward**	5′-ATA AGA ATG CGG CCG CAC CAT GGT GTT TTC GAT GTG GACA-3′
**TLR7 C-terminal Not I forward**	5′-ATA AGA ATG CGG CCG CGG AGC CAC CTT CTT TCT TGCC-3′
**HA Xho I reverse**	5′-CCG CTC GAG CGG TTA GGC GTA GTC TGG CAT GG-3′
**TLR7 N-terminal reverse (first step PCR)**	5′-AGT TTG AGC ATT AGG ACA AAAG-3′
**TLR7 N-terminal reverse (second step PCR),**	5′-CCG CTC GAG TTA GGC GTA GTC TGG CAC ATC ATA GGG GTA AGT TTG AGC AT-3′
**MHC class I leader sense**	5′-GGC CGC ACC ATG GTC CCG TGC ACG CTG CTC CTG CTG TTG GCA GCC GCC CTG GCT CCG ACT CAG ACC CGG GCC GGT ACC GC-3′
**MHC class I leader antisense**	5′-GGC CGC GGT CCG GCC CGG GTC TGA GTC GGA GCC AGG GCG GCT GCC AAC AGC AGG AGC AGC GTG CAC GGG ACC ATG GTGC-3
**primers TLR7 mutant sense**	5′-TTG CAA ACT TTA ACA CAG GAA ACA TTT GTG TCA-3′
**TLR7 mutant antisense**	5′-TGA CAC AAA TGT TTC CTG TGT TAA AGT TTG CAA-3′

The N-terminal insert was generated by two PCRs in order to add a HA-tag, using the first PCR product as template for the second PCR. In order to target the C-terminal constructs to the ER, the leader sequence from murine MHC class I was added by synthesis. The leader-coding oligonucleotide was subsequently inserted in front of the C-terminal previously cloned into pcDNA3.1. The full-length mTLR7 construct was mutagenized in its Asn 478 into Gln using the Quick-change mutagenesis kit (Stratagene).

### ^35^S-labelling of TLR7 protein, digestion assays and transfection in DCs

TLR7 pcDNA3.1 plasmid was transcribed and translated *in vitro* using TNT T7 quick Coupled Transcription/Translation system (Promega) and 10 µCi ^35^S-methionine (Perkin Elmer). Digestion assays were performed as previously described [16]. 10^6^ BM-DCs at day 6 were transfected with 1 µg of cDNA coding for TLR7-FL, -C-ter or -N-ter using the mouse DC Amaxa kit (Lonza, Germany). 48 h later, cells were harvested and stimulated with TLR agonists.

### Immunofluorescence

Cells were fixed with 4% paraformaldehyde for 10 min at RT, and quenched in 100 mM glycine for 20 min. Fixed cells were permeabilised and incubated with anti-HA (homemade) and anti-CD107a LAMP1 (eBIO1, clone D4B) antibodies in PBS-0.2% BSA-0.05% saponin. Immunofluorescence images were acquired on a Leica confocal microscope.

### Duolink

Duolink (OLINK) was performed according to the manufacturer's instructions. Briefly, DCs were grown on coverslips and then fixed in 4% paraformaldehyde for 10 min before permeabilization in PBS/0.05% saponin/0.2% BSA for 10 min. Cells were then blocked in 3% BSA/PBS and primary antibodies were incubated (anti-MyD88 and anti-TLR7). After washing the cells, PLA probes were added, followed by hybridization, ligation, and amplification for 90 min at 37°C. Nucleus (DAPI labelling) and MyD88-TLR7 interactions (green) were visualised after incubation with the detection solution. Slides were analyzed by confocal microscopy. Quantification of mean fluorescence using Image J software.

### Statistical analysis

Statistical significance was determined by unpaired *t*-test or two-way ANOVA.

## Supporting Information

Figure S1 **The innate inflammatory infiltrate of IAV-infected lungs was not altered by the absence of AEP.** (A) BAL fluid levels of IFN-α in wt or AEP^−/−^ mice before (NI), 4 d or 8 d post-viral infection. (n = 4 animals; graphs show mean ± SEM, * p<0.05). (B) IFN-β protein expression was quantified by quantitative real-time RT-PCR in total RNA extracted from lungs of wt or AEP^−/−^ mice before (NI), 4 d or 8 d post-viral infection. (n = 4–8 animals; graphs show mean ± SEM). (C) Flow cytometry analysis of single-cell suspensions of wt or AEP^−/−^ mice-BAL fluids before (NI), 4 d or 8 d post-viral infection. We analysed the presence of macrophages/monocytes (CD11b^+^/Gr1^Inter^), neutrophils (CD11b^+^/Gr1^high^) and dendritic cells (CD11b^+^/CD11c^+^) in BALs (n = 8 animals; graphs show mean ± SEM of two independent experiments). (D) Myeloperoxidase (MPO) activity measured in BAL fluids from wt or AEP^−/−^ mice 4 d and 8 d post-viral infection. (n = 8 animals; graphs show mean ± SEM of two independent experiments, ** p<0.01).
(TIF)

Figure S2 **Reduced TLR7 response in mice- or lung epithelial cells- lacking TLR7 infected with IAV virus.** (A) BAL fluid levels of cytokines (KC, IL-6, IL-12p40 and IFN-γ) in wt or TLR7^−/−^ mice before (NI), 4 d or 8 d after intranasal injection of IAV PR8 virus (100 pfu/mice). (n = 3–4 animals; graphs show mean ± SEM, * p<0.05, ** p<0.01). (B) IL-6 and RANTES secretion in supernatants of wt (white bars) or TLR7^−/−^ (gray bars) lung primary epithelial cells activated with 5 µg/mL of imiquimod or with the IAV PR8 (10 pfu) for 16 h or 24 h.
(TIF)

Figure S3 **Similar cross presentation of OVA in WT or AEP^−/−^ DCs with or without TLR3 stimulation.** Proliferation of OT-I T cells cultured with DCs from wt or AEP^−/−^ incubated with splenocytes from Balb/C mice (H-2^d^) electroporated with OVA and stimulated or not with 100 µg/mL poly(I∶C) or PR8 (10 pfu). Results are representative of two independent experiments.
(TIF)

Figure S4 **Cytokine production by AEP and cathepsin deficient pDCs, BMDCs upon TLR7 and TLR4 ligand stimuli.** (A) IFN-α secretion in supernatants of AEP^+/+^, AEP^−/−^ or TLR7^−/−^ pDCs activated with the IAV strain PR8 heat killed (HK) or live at a multiplicity of infection = 1 or 5 for 16 h or 24 h. (n = 2–3; mean ± SEM). (B, C) BMDCs from AEP^−/−^, CatB^−/−^, CatK^−/−^, CatL^−/−^, CatS^−/−^ mice (black bars) and from their wild type littermates (white bars) were stimulated with increasing concentrations of TLR agonist: imiquimod or resiquimod (B) for TLR7 and LPS for TLR4 (C) for 16 h and secretion of IL-6 were measured by ELISA. (n = 2–6, mean ± SEM). (D) Protease activities using specific substrates for CatB, CatB and CatL, CatK and CatS were measured in protein lysates of imiquimod stimulated DCs from wt (white symbols) and AEP-deficient mice (black symbols). (n = 2–3). (E) Protease activity of different cathepsins in total lysates from CatK^−/−^ and CatK^+/+^ BMDCs was measured using specific fluorescent substrates.
(TIF)

Figure S5 **Cytokine and co-stimulatory molecule expression in CatB^−/−^ and CatB^+/+^ DCs** ***in vivo*** **upon TLR7 sensing.** (A) FACS analysis of *in vivo* maturation of spleen CD11c^+^ cells 4 h after i.v. injection of 10 µg of imiquimod (left panel) or 1 µg of LPS (right panel) compared to no TLR ligand stimulation equivalent to 1. (B) IL-6 (left panel) and IL-12p40 (right panel) secretion were measured in serum of CatB^+/+^ or CatB^−/−^ mice 2 h after i.v. injection with imiquimod or PBS. (n = 7 animals for imiquimod; n = 2 animals for PBS; mean ± SEM for A and B).
(TIF)

Figure S6 **TLR7 is digested** ***in vitro*** **by AEP or cathepsins L or S.** *In vitro* transcription and translation of murine (A) or human (B) TLR7 FL followed by 2 h digestion of radiolabelled TLR7 FL with 15 U of rAEP and cathepsins (ND: non-digested). Data are representative of three experiments.
(TIF)

Figure S7 **TLR7 C-ter is functional and its generation is AEP-dependent.** (A) Immunodetection of TLR7 proteins in early (20 min) and late (120 min) phagosomes from wt BMDCs unstimulated or stimulated with 10 µg/ml of imiquimod. (B) TLR7 deficient DCs were transfected with cDNAs encoding for the empty vector (pcDNA3.1), TLR7 FL or C-ter TLR7 fragment. After 48 h, cells were stimulated with imiquimod for 16 h and IL-12p40 was measured in the supernatants. The amount of IL-12p40 produced by unstimulated cells was subtracted from imiquimod-stimulated cells. (Graphs show mean ± SEM, n = 3). (C) IL-6 and IL-12p40 secretion in TLR7^−/−^ BMDCs transfected with pcDNA3.1, FL or C-ter TLR7 fragment and stimulated with increasing concentrations of imiquimod for 16 h. (n = 4; mean ± SEM). (D) IL-12p40 secretion in TLR7^−/−^ BMDCs transfected with FL or N478Q TLR7 and stimulated with 5 µg/mL imiquimod for 16 h. (n = 4; mean ± SEM, * p<0.05).
(TIF)

Figure S8 **TLR7 C-ter fragment restores TLR7 response in AEP^−/−^ DCs and TLR7 stimulation induces acidic pH.** (A) AEP deficient (black bars) and wt (white bars) DCs were transfected with cDNAs encoding for the empty vector (pcDNA3.1), TLR7 FL or C-ter TLR7 fragment. After 48 h, cells were stimulated with imiquimod or LPS for 16 h and IL-6 was measured in the supernatants. The amount of IL-6 produced by unstimulated cells was substracted from imiquimod-stimulated cells. Graphs show mean ± SEM, n = 3, * p<0.05. (B) Kinetic of endo/lysosomal pH in AEP^+/+^ and AEP^−/−^ BMDCs in the presence or not of 10 µg/ml imiquimod and chased for different times. (n = 2–5; mean ± SEM, * p<0.05). (C, D) AEP activity in total lysate (C) or in phagosomes (D) from wt BMDCs treated or not with imiquimod (5 µg/mL) for the indicated time. (n = 3–4; mean ± SEM, ** p<0.01, *** p<0.001). (E) Protease activities using specific substrates for CatB, CatB and CatL, CatK and CatS were measured in protein lysates of imiquimod stimulated DCs from wt mice (n = 2–3; mean ± SEM, * p<0.05, ** p<0.01, *** p<0.001).
(TIF)

Figure S9 **Model for TLR7 processing and signaling.** Single stranded RNA of viral origin (IAV) or chemical compounds (imiquimod) are sensed by the intracellular receptor TLR7. After binding on the cell surface, IAV is internalized into endosomes where its genome is release in an acidic pH dependent fashion. Following TLR7 ligand stimulation, which induces a drop of pH in the endosomes and the recruitment of AEP, TLR7 translocates with the help of UNC93B1 from the ER to the endosomes. In the endosomes, TLR7 is cleaved in a C-terminal fragment where after a conformational change binds the adaptor molecule MyD88. This binding triggers the activation of NF-κB or IRF, their translocation to the nucleus, and subsequently the production and release of cytokines and chemokines in DCs.
(TIF)
