# Supplementary figures and images for: Asparagine Endopeptidase Controls Anti-Influenza Virus Immune Responses through TLR7 Activation
Source: PLoS Pathog. 2012 Aug 16;8(8):e1002841. doi: 10.1371/journal.ppat.1002841 (PMC3420946; doi:10.1371/journal.ppat.1002841)

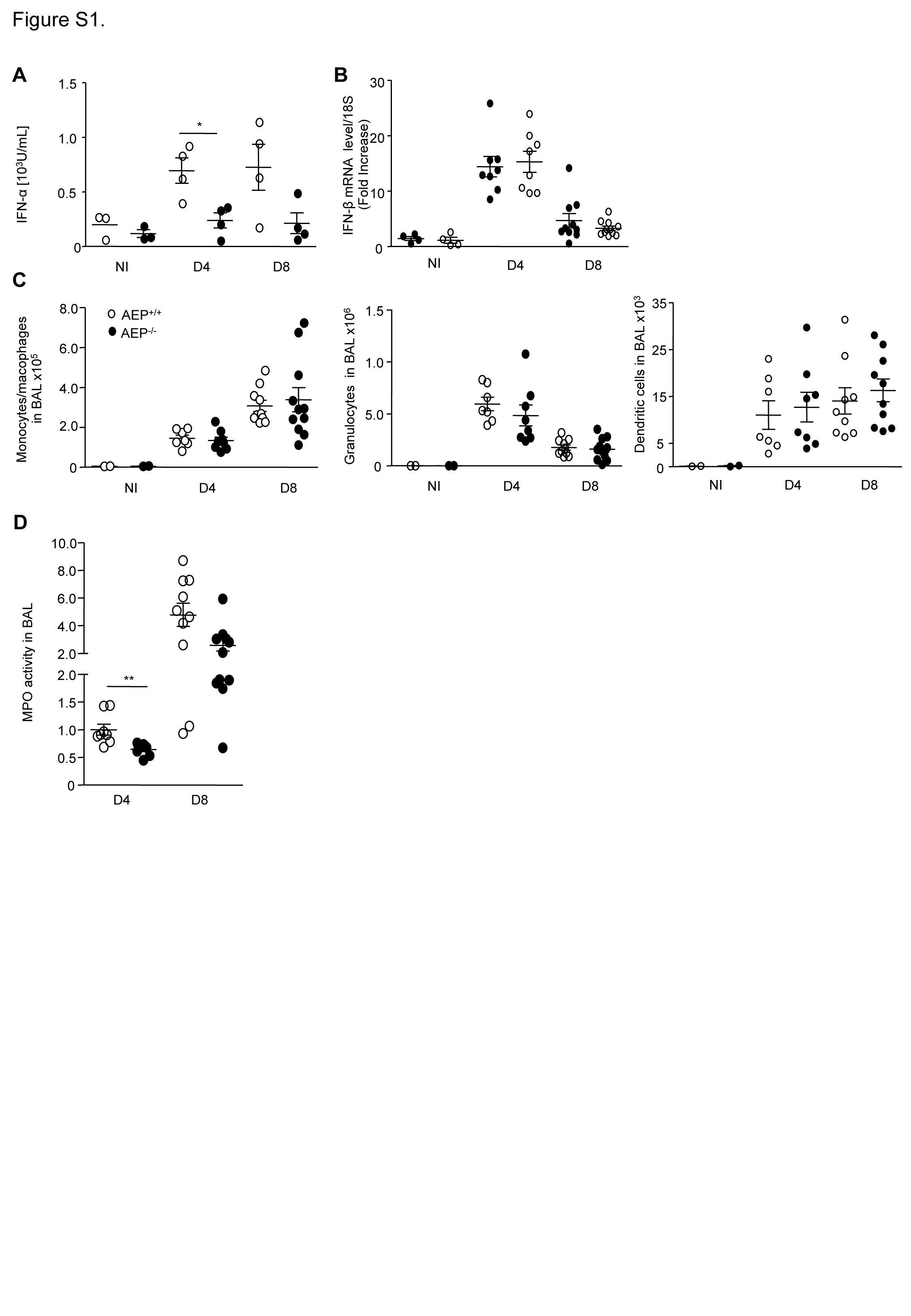

Supplement: Figure S1 — The innate inflammatory infiltrate of IAV-infected lungs was not altered by the absence of AEP. (A) BAL fluid levels of IFN-α in wt or AEP−/− mice before (NI), 4 d or 8 d post-viral infection. (n = 4 animals; graphs show mean ± SEM, * p<0.05). (B) IFN-β protein expression was quantified by quantitative real-time RT-PCR in total RNA extracted from lungs of wt or AEP−/− mice before (NI), 4 d or 8 d post-viral infection. (n = 4–8 animals; graphs show mean ± SEM). (C) Flow cytometry analysis of single-cell suspensions of wt or AEP−/− mice-BAL fluids before (NI), 4 d or 8 d post-viral infection. We analysed the presence of macrophages/monocytes (CD11b+/Gr1Inter), neutrophils (CD11b+/Gr1high) and dendritic cells (CD11b+/CD11c+) in BALs (n = 8 animals; graphs show mean ± SEM of two independent experiments). (D) Myeloperoxidase (MPO) activity measured in BAL fluids from wt or AEP−/− mice 4 d and 8 d post-viral infection. (n = 8 animals; graphs show mean ± SEM of two independent experiments, ** p<0.01). (TIF) [file ppat.1002841.s001.tif]

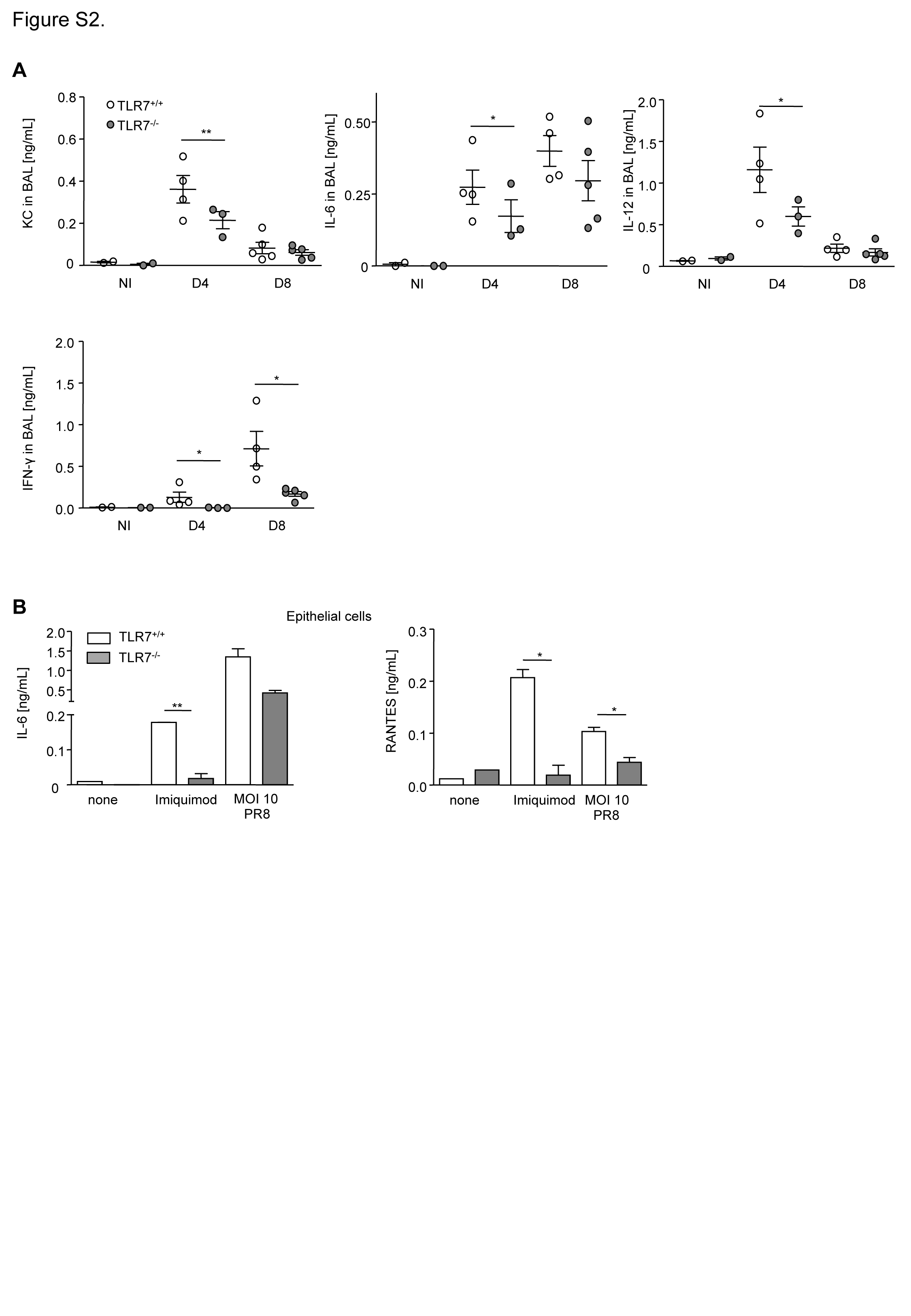

Supplement: Figure S2 — Reduced TLR7 response in mice- or lung epithelial cells- lacking TLR7 infected with IAV virus. (A) BAL fluid levels of cytokines (KC, IL-6, IL-12p40 and IFN-γ) in wt or TLR7−/− mice before (NI), 4 d or 8 d after intranasal injection of IAV PR8 virus (100 pfu/mice). (n = 3–4 animals; graphs show mean ± SEM, * p<0.05, ** p<0.01). (B) IL-6 and RANTES secretion in supernatants of wt (white bars) or TLR7−/− (gray bars) lung primary epithelial cells activated with 5 µg/mL of imiquimod or with the IAV PR8 (10 pfu) for 16 h or 24 h. (TIF) [file ppat.1002841.s002.tif]

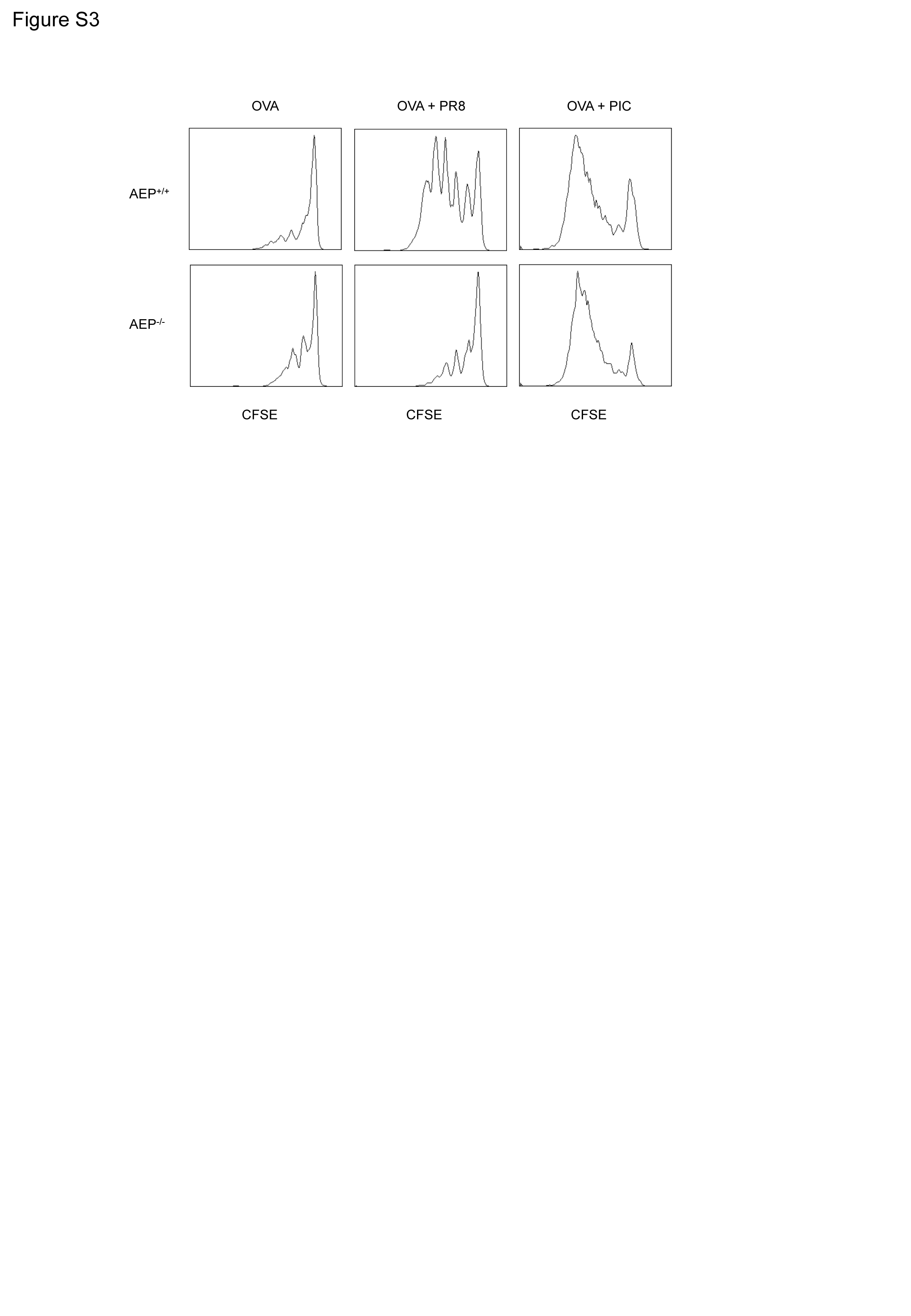

Supplement: Figure S3 — Similar cross presentation of OVA in WT or AEP−/− DCs with or without TLR3 stimulation. Proliferation of OT-I T cells cultured with DCs from wt or AEP−/− incubated with splenocytes from Balb/C mice (H-2d) electroporated with OVA and stimulated or not with 100 µg/mL poly(I∶C) or PR8 (10 pfu). Results are representative of two independent experiments. (TIF) [file ppat.1002841.s003.tif]

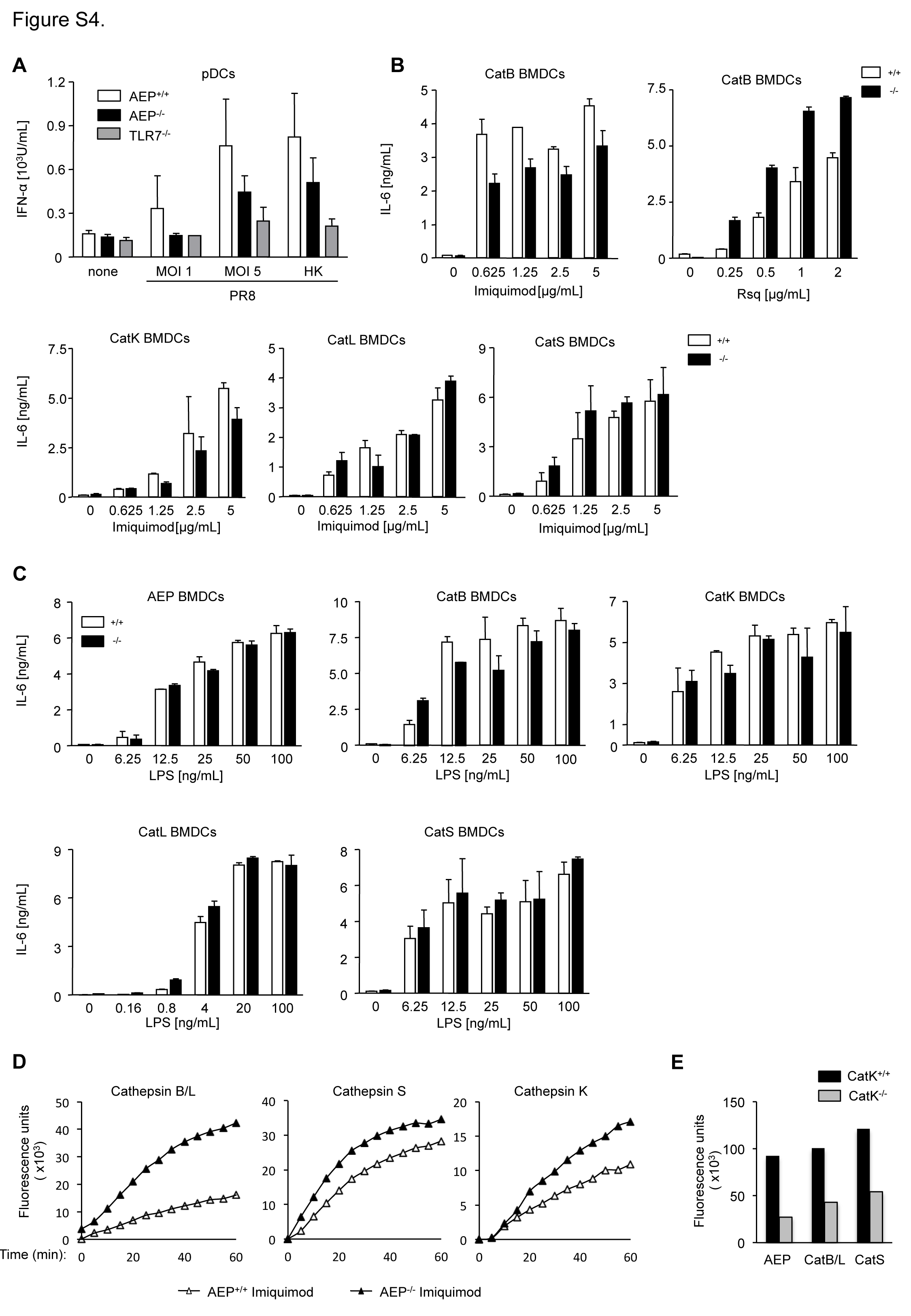

Supplement: Figure S4 — Cytokine production by AEP and cathepsin deficient pDCs, BMDCs upon TLR7 and TLR4 ligand stimuli. (A) IFN-α secretion in supernatants of AEP+/+, AEP−/− or TLR7−/− pDCs activated with the IAV strain PR8 heat killed (HK) or live at a multiplicity of infection = 1 or 5 for 16 h or 24 h. (n = 2–3; mean ± SEM). (B, C) BMDCs from AEP−/−, CatB−/−, CatK−/−, CatL−/−, CatS−/− mice (black bars) and from their wild type littermates (white bars) were stimulated with increasing concentrations of TLR agonist: imiquimod or resiquimod (B) for TLR7 and LPS for TLR4 (C) for 16 h and secretion of IL-6 were measured by ELISA. (n = 2–6, mean ± SEM). (D) Protease activities using specific substrates for CatB, CatB and CatL, CatK and CatS were measured in protein lysates of imiquimod stimulated DCs from wt (white symbols) and AEP-deficient mice (black symbols). (n = 2–3). (E) Protease activity of different cathepsins in total lysates from CatK−/− and CatK+/+ BMDCs was measured using specific fluorescent substrates. (TIF) [file ppat.1002841.s004.tif]

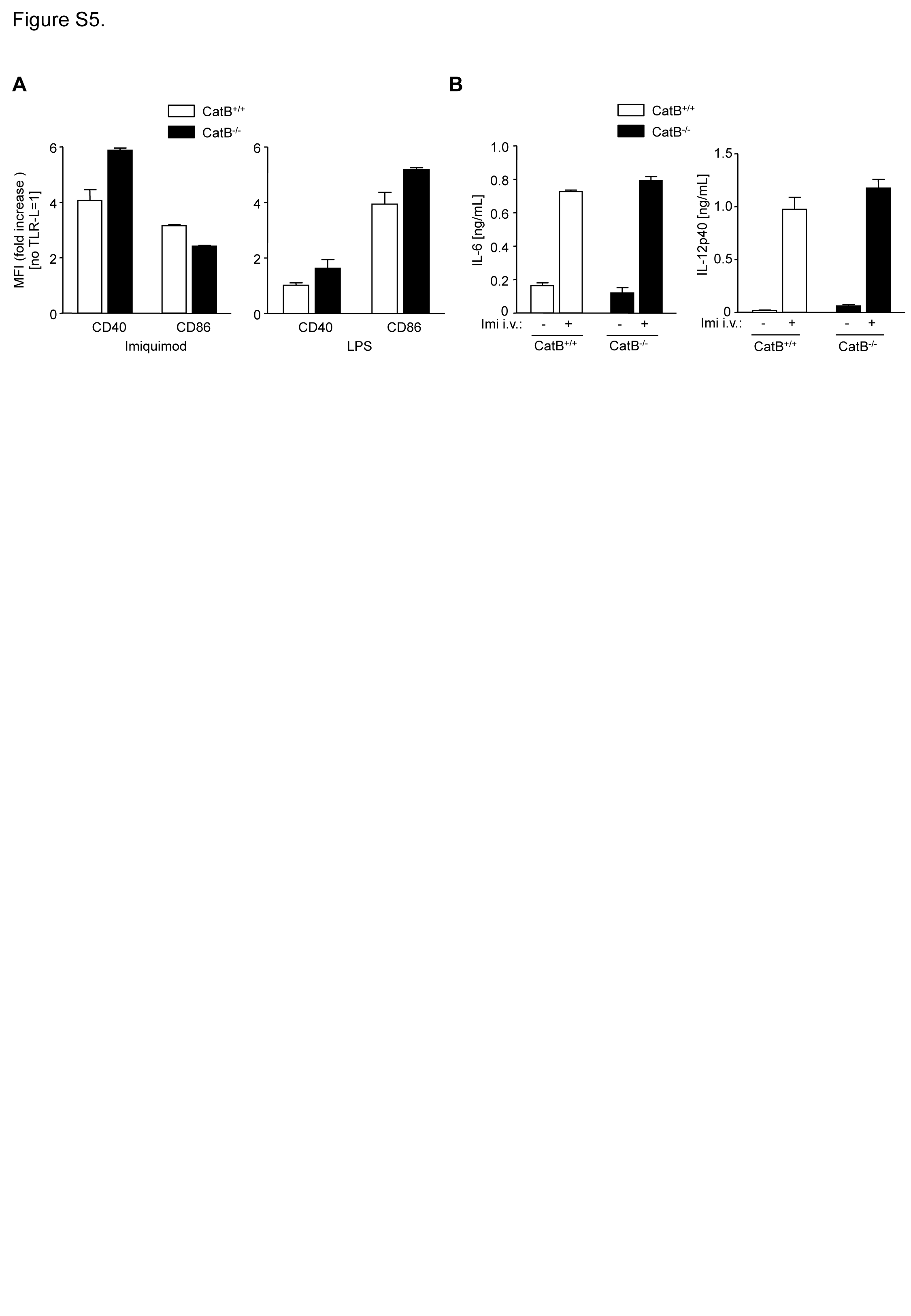

Supplement: Figure S5 — Cytokine and co-stimulatory molecule expression in CatB−/− and CatB+/+ DCs in vivo upon TLR7 sensing. (A) FACS analysis of in vivo maturation of spleen CD11c+ cells 4 h after i.v. injection of 10 µg of imiquimod (left panel) or 1 µg of LPS (right panel) compared to no TLR ligand stimulation equivalent to 1. (B) IL-6 (left panel) and IL-12p40 (right panel) secretion were measured in serum of CatB+/+ or CatB−/− mice 2 h after i.v. injection with imiquimod or PBS. (n = 7 animals for imiquimod; n = 2 animals for PBS; mean ± SEM for A and B). (TIF) [file ppat.1002841.s005.tif]

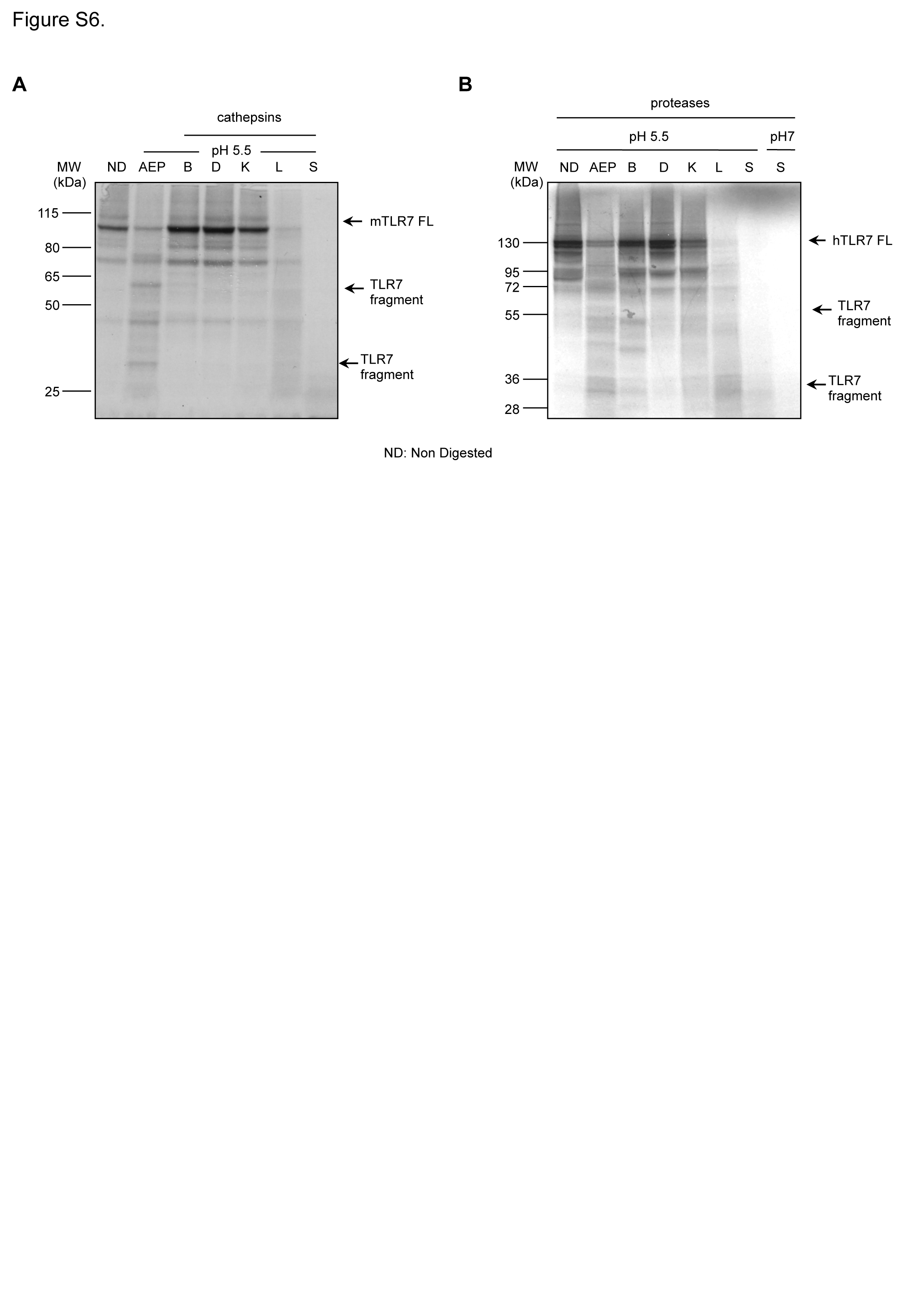

Supplement: Figure S6 — TLR7 is digested in vitro by AEP or cathepsins L or S. In vitro transcription and translation of murine (A) or human (B) TLR7 FL followed by 2 h digestion of radiolabelled TLR7 FL with 15 U of rAEP and cathepsins (ND: non-digested). Data are representative of three experiments. (TIF) [file ppat.1002841.s006.tif]

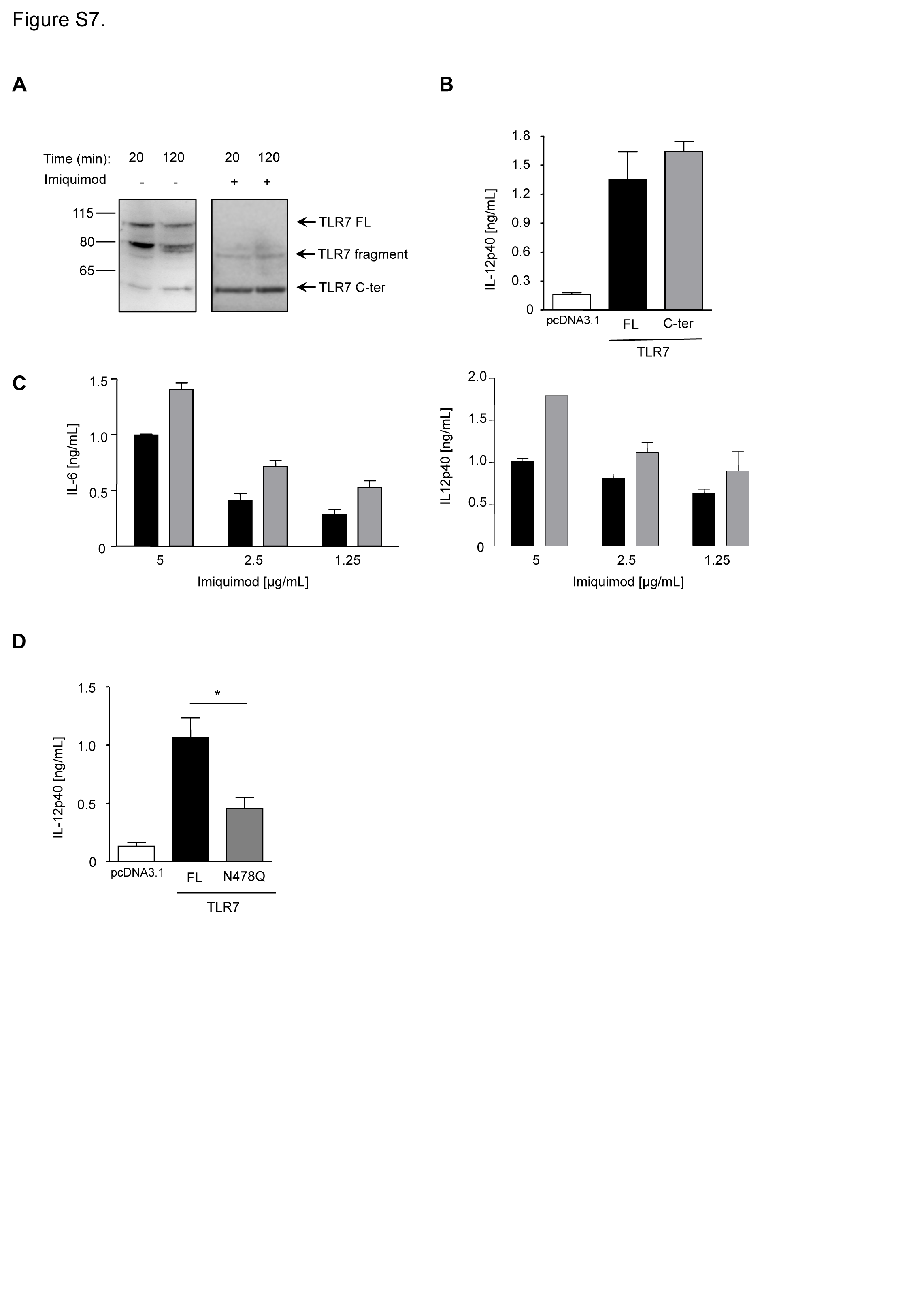

Supplement: Figure S7 — TLR7 C-ter is functional and its generation is AEP-dependent. (A) Immunodetection of TLR7 proteins in early (20 min) and late (120 min) phagosomes from wt BMDCs unstimulated or stimulated with 10 µg/ml of imiquimod. (B) TLR7 deficient DCs were transfected with cDNAs encoding for the empty vector (pcDNA3.1), TLR7 FL or C-ter TLR7 fragment. After 48 h, cells were stimulated with imiquimod for 16 h and IL-12p40 was measured in the supernatants. The amount of IL-12p40 produced by unstimulated cells was subtracted from imiquimod-stimulated cells. (Graphs show mean ± SEM, n = 3). (C) IL-6 and IL-12p40 secretion in TLR7−/− BMDCs transfected with pcDNA3.1, FL or C-ter TLR7 fragment and stimulated with increasing concentrations of imiquimod for 16 h. (n = 4; mean ± SEM). (D) IL-12p40 secretion in TLR7−/− BMDCs transfected with FL or N478Q TLR7 and stimulated with 5 µg/mL imiquimod for 16 h. (n = 4; mean ± SEM, * p<0.05). (TIF) [file ppat.1002841.s007.tif]

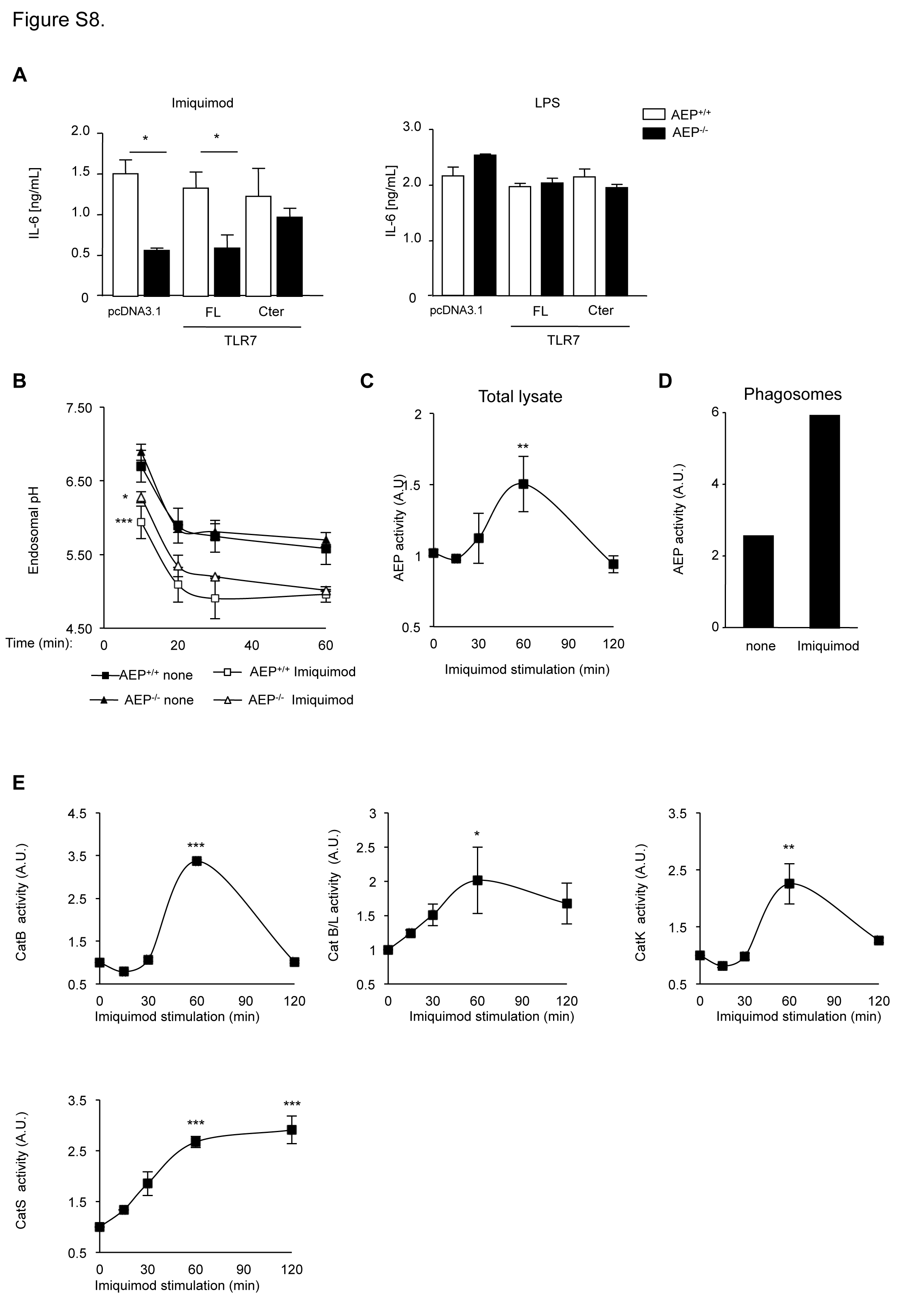

Supplement: Figure S8 — TLR7 C-ter fragment restores TLR7 response in AEP−/− DCs and TLR7 stimulation induces acidic pH. (A) AEP deficient (black bars) and wt (white bars) DCs were transfected with cDNAs encoding for the empty vector (pcDNA3.1), TLR7 FL or C-ter TLR7 fragment. After 48 h, cells were stimulated with imiquimod or LPS for 16 h and IL-6 was measured in the supernatants. The amount of IL-6 produced by unstimulated cells was substracted from imiquimod-stimulated cells. Graphs show mean ± SEM, n = 3, * p<0.05. (B) Kinetic of endo/lysosomal pH in AEP+/+ and AEP−/− BMDCs in the presence or not of 10 µg/ml imiquimod and chased for different times. (n = 2–5; mean ± SEM, * p<0.05). (C, D) AEP activity in total lysate (C) or in phagosomes (D) from wt BMDCs treated or not with imiquimod (5 µg/mL) for the indicated time. (n = 3–4; mean ± SEM, ** p<0.01, *** p<0.001). (E) Protease activities using specific substrates for CatB, CatB and CatL, CatK and CatS were measured in protein lysates of imiquimod stimulated DCs from wt mice (n = 2–3; mean ± SEM, * p<0.05, ** p<0.01, *** p<0.001). (TIF) [file ppat.1002841.s008.tif]

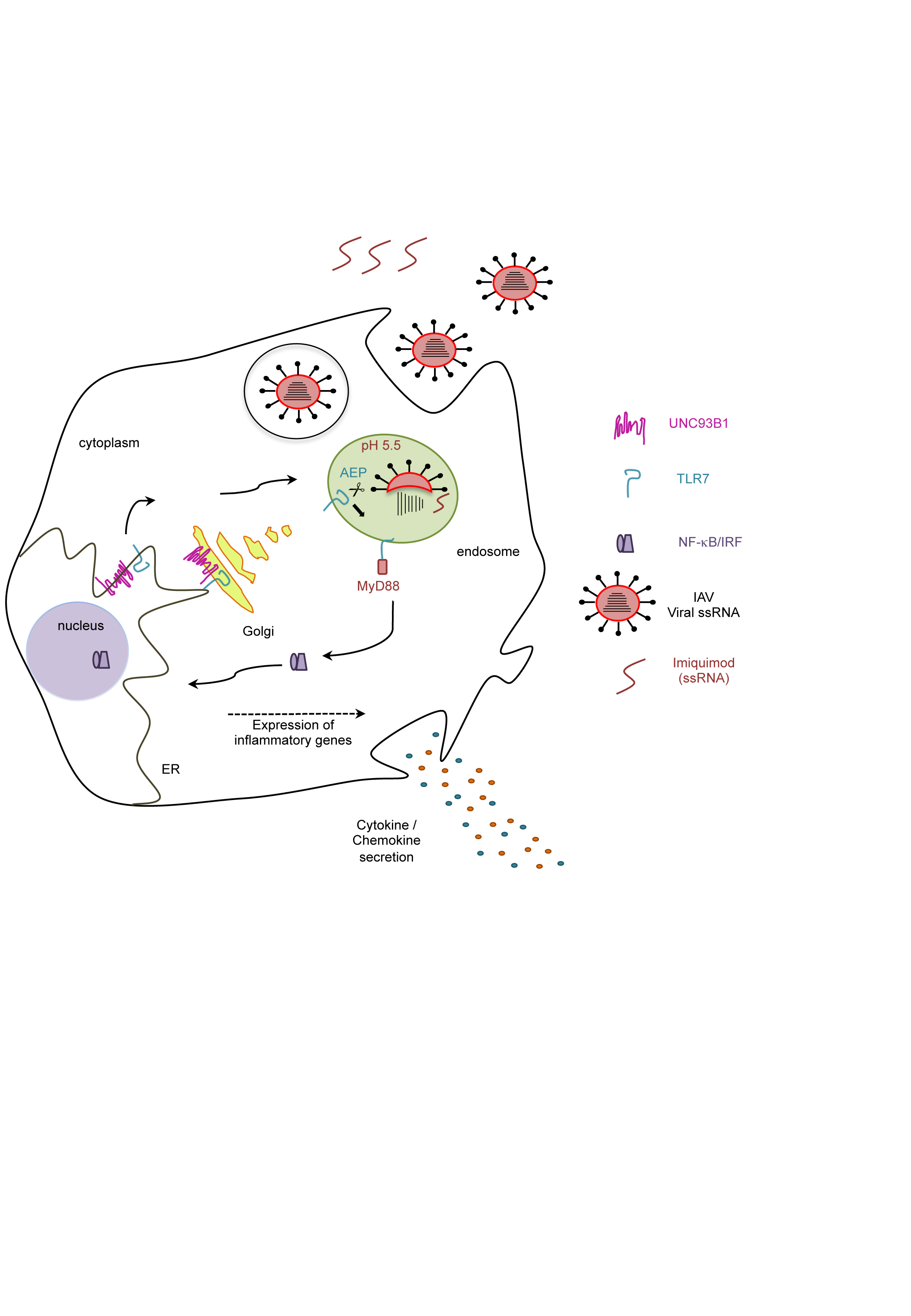

Supplement: Figure S9 — Model for TLR7 processing and signaling. Single stranded RNA of viral origin (IAV) or chemical compounds (imiquimod) are sensed by the intracellular receptor TLR7. After binding on the cell surface, IAV is internalized into endosomes where its genome is release in an acidic pH dependent fashion. Following TLR7 ligand stimulation, which induces a drop of pH in the endosomes and the recruitment of AEP, TLR7 translocates with the help of UNC93B1 from the ER to the endosomes. In the endosomes, TLR7 is cleaved in a C-terminal fragment where after a conformational change binds the adaptor molecule MyD88. This binding triggers the activation of NF-κB or IRF, their translocation to the nucleus, and subsequently the production and release of cytokines and chemokines in DCs. (TIF) [file ppat.1002841.s009.tif]
